# Targeted error-suppressed quantification of circulating tumor DNA using semi-degenerate barcoded adapters and biotinylated baits

**DOI:** 10.1038/s41598-017-10269-2

**Published:** 2017-09-05

**Authors:** Miguel Alcaide, Stephen Yu, Jordan Davidson, Marco Albuquerque, Kevin Bushell, Daniel Fornika, Sarah Arthur, Bruno M. Grande, Suzan McNamara, Mathilde Couetoux du Tertre, Gerald Batist, David G. Huntsman, Luca Cavallone, Adriana Aguilar, Mark Basik, Nathalie A. Johnson, Rebecca J. Deyell, S. Rod Rassekh, Ryan D. Morin

**Affiliations:** 1 Department of Molecular Biology and Biochemistry, Simon Fraser University, Burnaby, BC Canada; 2 Quebec Clinical Research Organization in Cancer (Q-CROC), Exactis Innovation and the Segal Cancer Centre, Montreal, QC Canada; 3 Department of Molecular Oncology, British Columbia Cancer Agency, Vancouver, BC Canada; 4 Department of Pathology and Laboratory Medicine and Department of Obstetrics and Gynecology, University of British Columbia, Vancouver, BC Canada; 5 Department of Medicine, Jewish General Hospital, Montreal, Quebec Canada; 6 Division of Oncology, Hematology and Bone Marrow Transplant, British Columbia Children’s Hospital and University of British Columbia, Vancouver, British Columbia Canada

## Abstract

Ultrasensitive methods for rare allele detection are critical to leverage the full potential offered by liquid biopsies. Here, we describe a novel molecular barcoding method for the precise detection and quantification of circulating tumor DNA (ctDNA). The major benefits of our design include straightforward and cost-effective production of barcoded adapters to tag individual DNA molecules before PCR and sequencing, and better control over cross-contamination between experiments. We validated our approach in a cohort of 24 patients with a broad spectrum of cancer diagnoses by targeting and quantifying single-nucleotide variants (SNVs), indels and genomic rearrangements in plasma samples. By using personalized panels targeting *a priori* known mutations, we demonstrate comprehensive error-suppression capabilities for SNVs and detection thresholds for ctDNA below 0.1%. We also show that our semi-degenerate barcoded adapters hold promise for noninvasive genotyping in the absence of tumor biopsies and monitoring of minimal residual disease in longitudinal plasma samples. The benefits demonstrated here include broad applicability, flexibility, affordability and reproducibility in the research and clinical settings.

## Introduction

Liquid biopsies offer an enormous potential for the noninvasive genotyping and real-time monitoring of solid tumor disease status^1, 2^ and may facilitate a more comprehensive genomic profiling in cancers with substantial tumor heterogeneity or multiple metastases^3, 4^. Clinical application of liquid biopsies to inform molecular-based risk stratification and guide therapeutic intervention strategies may help reduce morbidity and overall costs, particularly for cancers where obtaining repeated tumor biopsies is challenging or unsafe^5, 6^. A plethora of research reinforces the utility of circulating tumor DNA (ctDNA) to non-invasively estimate tumor burden, stratify patients to the best treatment according to the presence of actionable mutations, ascertain response to such treatments, and promptly detect emergent somatic mutations associated with therapeutic resistance^6–10^. The amount of tumor-derived mutant DNA circulating in biofluids, however, can be extremely limiting in many patients. This is more pronounced in certain cancer types and early stages or when residual disease persists after surgery^11–14^. Substantial efforts have been therefore aimed at the development of sophisticated molecular approaches for the accurate detection and quantification of ctDNA^15, 16^.

Ultrasensitive approaches such as digital PCR (dPCR), and less commonly, CypherSeq, have proven particularly efficient concerning rare allele detection^17–19^. Alternative approaches combine either PCR amplicon^20^ or targeted hybrid capture with biotinylated baits followed by next-generation sequencing. Commonly overlooked benefits of ligation-based capture of cell-free DNA (cfDNA), for instance CAPP-seq^7^, are that PCR-based approaches may fail to successfully report ctDNA fragments lacking at least one priming site and can also be more negatively impacted by biological contaminants from cell lysis. Another key benefit is the potential scalability to larger regions or to span panels of mutations. Barriers in adapting capture-based approaches to ctDNA included restrictions in recovery of cfDNA molecules and relatively lower specificity due to errors introduced during library preparation and sequencing^16, 21^. Improvements in library construction and molecular barcoding strategies have addressed these by including error-suppression methods that may extend the limit of detection to compete with dPCR and even potentially provide higher specificity^19, 21–23^.

**Figure 1:**
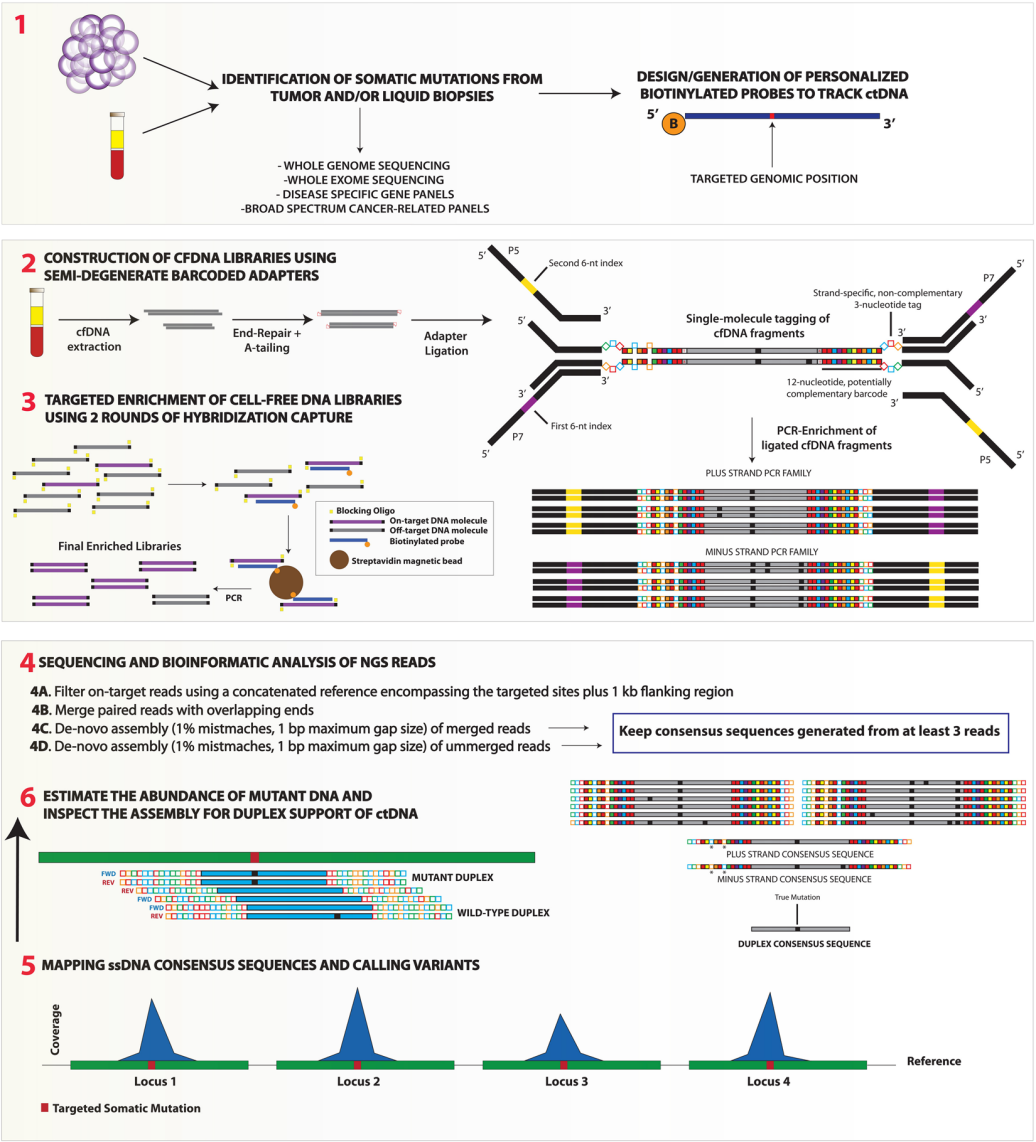
Overview of the experimental workflow to track ctDNA in cancer patients using semi-degenerate barcoded adapters and personalized panels of biotinylated baits. Biotinylated baits targeting somatic mutations previously identified via the sequencing of tumor/liquid biopsies and matched normal DNA samples are generated “in-house” or ordered from commercial manufacturers (1). Next, libraries are built using the cfDNA isolated from liquid biopsy specimens (2). End-repaired and A-tailed cfDNA fragments are ligated with partially complementary double-stranded barcoded adapters and then PCR-amplified with 6-nucleotide dual-indexed primers that provide P5 and P7 Illumina adapter sequences. Our custom adapters are comprised of the annealing of two oligonucleotides that harbor non-complementary tri-nucleotide tags for either the plus (5′–3′) or the minus (3′–5′) strand. Different nucleotides within the fixed tags are represented by colours (A:red; C:blue; T:green; G:orange). This adapter design also includes a semi-degenerate and potentially complementary 12-nucleotide barcode sequence ((5′-WSMRWSYWKMWW-3′) in plus strand; (5′-WWKMWRSWYKSW-3′) in minus strand)). During the annealing of the two oligonucleotides a perfect complementary match can occur (right adapter) but, more commonly, hybridizations include annealing mispairings (left adapter). Solid red squares represent either A-T or T-A base pairings (W vs W); solid yellow squares represent either G-C or C-G base pairings (S vs S); solid blue squares represent either C-G or A-T base pairings (M vs K); orange squares represent G-C or A-T (R vs Y); solid green squares represent C-G or T-A base pairings (Y vs R) and solid violet squares represent G-C or T-A base pairings (K vs M). Annealing mispairings (see left adapter) are denoted by the presence of the same base at equivalent positions in both strands. Libraries are then subjected to two rounds (ideally) of hybridization capture using personalized panels of biotinylated baits and final enriched libraries are sequenced on Illumina platforms (3). The bioinformatic analysis of the NGS reads involves the filtering of on-target reads, merging of paired reads with overlapping ends and generation of consensus sequences according to a *de-novo* assembly approach that allows for a maximum of 1% mismatches and maximum gap size of 1 bp (4). In essence, the two parental strands derived from every single cfDNA molecule generate independent PCR families. Consensus sequences are generated from each PCR family with at least three independent reads. Consensus sequences from independent strand orientations are considered to derive from the same cfDNA molecule if they share the same start/end positions in the reference sequence and if they do not show more than 2 mismatches in the last 6 semi-degenerate barcode positions flanking the ligation site. Duplex sequencing allows correcting any strand-specific errors or variants deriving from DNA damage. After sequencing, solid red squares represent W degenerate positions (i.e. either A or T); solid yellow squares = S; solid blue squares = M; solid orange squares = R; solid green squares = Y; solid violate squares = K). Annealing mismatches are denoted by white squares and indicated by asterisks. Black squares represent discrepancies with respect to the reference sequence. Consensus sequences are finally mapped against the reference sequence (5) and targeted genomic positions are screened for duplex support of ctDNA and its abundance (6) Only variants independently supported by the consensus sequences of both parental strands are considered high-confidence.

Here, we describe a straightforward and cost-effective single-molecule molecular barcoding strategy that also offers improved control over cross-contamination. Our method relies on semi-degenerate barcoded adapters (Fig. 1) and has been evaluated on a series of liquid biopsies drawn from 24 patients with a broad spectrum of cancer diagnoses by using different targeted enrichment strategies followed by next-generation sequencing (Table 1). The main goals of the present study can be summarized as follows i) evaluate the performance of our barcoded adapters with respect to standard sequencing adapters ii) assess the molecular tagging properties derived from the use of our adapters at both the single-stranded and double-stranded DNA level iii) investigate the error-suppression potential of sequencing methods relying on our adapter constructs and iv) test our approach on liquid biopsies drawn from different patients using personalized panels of biotinylated baits and pre-designed disease-specific or “broad-spectrum” gene panels in cases with and without *a priori* information on previously identified somatic mutations. We show that sequencing of cfDNA libraries enriched with personalized baits are highly amenable to duplex sequencing^22^, hence addressing a limitation that has thus far impeded the full power of error-suppression methods when sequencing larger genomic regions due to low recovery efficiencies of DNA fragments represented by the two parental strands^21, 23^. Through direct detection of mutations in a gene panel, we demonstrate that our method also holds promise for the noninvasive genetic profiling and monitoring of solid tumors without representing a substantial increase in overall costs.

**Table 1:** Disease diagnosis and clinical enrollment of the 24 patients investigated in this study.

Patient ID	Disease Diagnosis	Clinical Trial	DNA Baits	OT Reads
NB-pt01	NEUROBLASTOMA	POG	Pt-Specif. (2x)	153 K, 201 K, 279 K 250 K, 271 K, 412 K
NB-pt02	NEUROBLASTOMA	POG	Pt-Specif. (1X)	1 K
NB-pt03	NEUROBLASTOMA	POG	Pt-Specif. (1X)	14 K
NB-pt04	NEUROBLASTOMA	POG	Pt-Specif. (2X)	1,400 K
OVC-pt01	OVARIAN GRANULOSA	POG	Pt-Specif. (2X)	2,400 K (V1); 1,400 K (V2)
OVC-pt02	OVARIAN CARCINOMA	POG	Pt-Specif. (2X)	1,800 K
OSS-pt01	OSTEOSARCOMA	POG	Pt-Specif. (2X)	450 K
OSS-pt02	OSTEOSARCOMA	POG	Pt-Specif. (1X)	20 K
NMC-pt01	NUT MIDLINE CARCINOMA	POG	Pt-Specif. (1X)	3 K
CPG-pt01	CRANIOPHARYNGIOMA	POG	Pt-Specif. (2X)	320 K
PIB-pt01	PINEOBLASTOMA	POG	Pt-Specif. (2X)	2,500 K
IFB-pt01	INFANTILE FIBROSARCOMA	POG	Pt-Specif. (2X)	1,280 K
ASL-pt01	ANGIOSARCOMA OF LIVER	POG	Pt-Specif. (1X)	77 K
SAR-pt01	SARCOMA	POG	Pt-Specif. (1X)	1 K
ESR-pt01	EWING SARCOMA	POG	Pan-Cancer. (1X)	400 K
MGC-pt01	MALIGNANT GRANULLAR CELL TUMOUR	POG	Pt-Specif. (2X)	770 K
HGL-pt01	HODGKIN LYMPHOMA	POG	Pan-Cancer + Dis-Specif. (1X)	2,000 K
DLBCL-pt01	DIFFUSE LARGE B-CELL LYMPHOMA	POG	Pt-Specif. (2X)	120 K
DLBCL-pt015	DIFFUSE LARGE B-CELL LYMPHOMA	Q-CROC-02	Dis-Specif. (1X)	1,600 K
ALL-pt01	ACUTE LYMPHOBLASTIC LEUKEMIA	POG	Pan-Cancer	650 K
CCR-pt029	COLORECTAL CANCER	Q-CROC-01	Pan-Cancer	4,800 K
CCR-pt049	COLORECTAL CANCER	Q-CROC-01	Pan-Cancer	3,600 K
Neo-02	BREAST CANCER	Q-CROC-03	*TP53*	4,500 K
Neo-027	BREAST CANCER	Q-CROC-03	*TP53*	2,400 K

This table shows whether the enrichment of the cell-free DNA was accomplished using patient-specific sets of biotinylated DNA baits (Pt-Specif.), panels of DNA probes spanning the coding regions of a single gene (*TP53*), disease-specific panels (Dis-Specif.) or broad panels of probes targeting several cancer-related genes (Pan-Cancer,see methods). The “DNA baits” column also indicates whether a single (1x) or two rounds of hybridization capture (2x) were carried out during each corresponding targeted enrichment experiment. The approximate number of on-target reads obtained for each patient is shown in the far right column (K = 10^3^). More than one value for patient NB-pt01 relates to the different library replicates built from the plasma of this patient (top row values: libraries built with barcoded adapters; bottom row values: libraries built with standard sequencing adapters).

## Results

### Recovery efficiencies of cfDNA molecules with semi-degenerate barcoded adapters

One of the most important factors determining detection limits of ctDNA is an efficient recovery of cfDNA fragments that are amenable to detection by the assay. We conducted a comparison between library triplicates that were built with either our semi-degenerate barcoded adapters or standard Y-shaped sequencing adapters from the plasma of a pediatric patient diagnosed with metastatic neuroblastoma (NB-pt01). The six libraries were individually indexed, pooled and simultaneously enriched using five biotinylated baits targeting *a priori* known somatic mutations, as revealed by whole genome sequencing of tumor biopsy and matched germline DNA samples. Overall, the average number of unique molecules covering each of the five targeted genomic sites was slightly higher, but not statistically different, in the three library replicates built with our barcoded adapters (404.2 ± 76.7 S.D.; 391.4 ± 66.4 S.D., respectively; two-tailed p-value = 0.19, Table 2). Although the variant allele frequencies (VAF) did vary among triplicates, particularly at the lower levels, this can be explained by Poisson variation and we found no significant differences between the averaged VAF estimated through the two sets of triplicates (Table 2). All libraries were subjected to the same bioinformatic pipeline (see methods) and we exclusively relied on mapping coordinate information to count unique DNA molecules. Although our molecular barcodes allow differentiating several molecules sharing exactly the same mapping coordinates we did not leverage barcoding information for this analysis because standard sequencing adapters do not offer that possibility. Given the amount of DNA entering library construction for each library replicate (2.3 ng, equivalent to 697 human haploid genomes/copies per locus if we assume 3.3 pg per haploid human genome) and the number of unique DNA molecules retrieved at each of the five targeted locus, our data suggests, roughly, recovery efficiencies for cfDNA ranging between 56–70% and 51–75% when using either barcoded or standard sequencing adapters, respectively. One locus was less efficiently enriched in both cases: 32–42% and 38–44%. (Table 2), likely reflecting differences in hybridization capture efficiency among baits. It must be noted that the number of unique DNA molecules retrieved in these relatively low diversity cfDNA libraries should be actually higher given that no molecular barcoding information to disentangle mapping coordinate collisions was considered for this particular analysis (see the end of next section).

**Table 2:** Number of unique cfDNA molecules mapping to five independent genomic positions (Loc1 to 5; based on unique mapping coordinates of library fragments) in the three library replicates (R1, R2, R3) that were built with either semi-degenerate barcoded adapters (BarAd) or standard sequencing adapters (StdAd) from the same amount of input cfDNA (2.3 ng) in patient NB-pt01.

	Loc1	Loc2	Loc3	Loc4	Loc5
BarAd - R1	280 (0.379)	394 (0.076)	457 (0.543)	418 (0.029)	396 (0.215)
BarAd - R2	228 (0.338)	411 (0.078)	469 (0.559)	450 (0.062)	460 (0.171)
BarAd - R3	294 (0.259)	439 (0.032)	492 (0.583)	438 (0.046)	437 (0.158)
**BarAd - Avg**	**267.33 (0.325)**	**414.66 (0.062)**	**472.66 (0.562)**	**435.33 (0.046)**	**431 (0.181)**
StdAd - R1	266 (0.349)	358 (0.047)	470 (0.521)	413 (0.041)	396 (0.186)
StdAd - R2	293 (0.307)	386 (0.054)	520 (0.544)	405 (0.039)	408 (0.200)
StdAd - R1	305 (0308)	381 (0.063)	447 (0.544)	412 (0.034)	411 (0.200)
**StdAd - Avg**	**288 (0.321)**	**375 (0.055)**	**479 (0.536)**	**410 (0.038)**	**405 (0.195)**
Two-tailed p-value	0.47	0.054	0.84	0.079	0.22

Estimated allele frequencies for mutant DNA circulating in the plasma are indicated in parentheses. For each triplicate series, one of the rows (in bold) shows average values. We did not find statistically significant differences concerning recovery efficiencies of cfDNA when comparing the number of unique molecules retrieved by either barcoded or standard sequencing adapters (bottom row). Genomic coordinates for the five single-nucleotide variants targeted in this patient are provided in Table S1.

### Molecular barcoding and consensus sequencing of individual DNA strands

Our adapters were designed to include a strand-specific tri-nucleotide tag and 12-nucleotide semi-degenerate barcode, which are both sequenced as part of the insert DNA and together allow each strand of a DNA duplex to be individually indexed prior to PCR and sequencing (Fig. 1, Supplemental File 1). Since some barcode sequences can be chemically synthesized more efficiently or generate adapters that ligate more proficiently, we first evaluated the barcoding complexity of single stranded DNA molecules (ssDNA) across cfDNA libraries built from the plasma of two cancer patients: OSS-Pt01 and OVC-Pt01 (V2). OSS-Pt01 was diagnosed with osteosarcoma and showed small metastatic pulmonary lesions. OVC-Pt01 was diagnosed with stage IV granulosa cell tumor of the ovary. These libraries were enriched during two rounds of hybridization capture by means of two personalized panels of biotinylated DNA baits (N = 4 and N = 6, respectively) targeting *a priori* known somatic mutations. We used our own custom DNA baits (see methods and Figure S1) in patient OSS-pt01 and ordered XGen**©** Lockdown**©** probes (Integrated DNA Technologies) for patient OVC-pt01. Targeted hybridization capture with in-house generated biotinylated baits performed well. Here, we obtained 48.85% on-target reads for OSS-pt01, versus 47.11% on-target reads for OVC-pt01 (V2) (see Figure S2).

**Figure 2:**
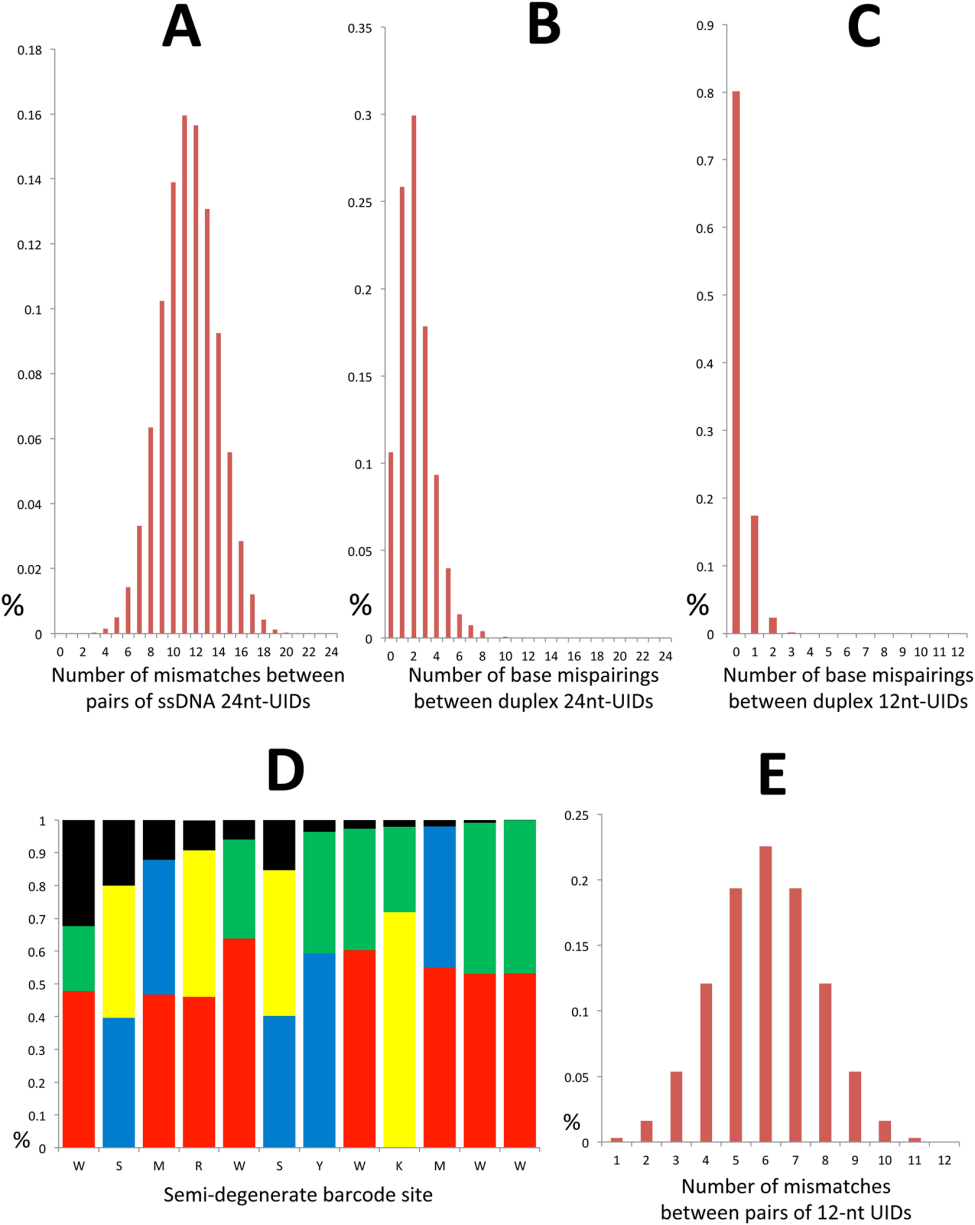
(**A**) Distribution of the number of nucleotide differences between two random unique molecule identifiers (UIDs) attached to one of the parental strands of dsDNA molecules. (**B**) Distribution of the number of base mispairings or annealing artifacts along the semi-degenerate barcoded region that arise during adapter annealing. (**C**) Annealing artifacts are less common within the last six nucleotides of each barcode (i.e. those preceding the ligation site). (**D**) Distribution of base composition (consensus A: red; consensus C: blue; consensus G: yellow; consensus T: green) and frequency of annealing artifacts (black bars) across every position of the 12-nucleotide semi-degenerate barcode of each adapter molecule. Some of the positions show skewed base ratios that can be attributed to the automated mixing method for randomization during manufacturing. Better ratios for certain semi-degenerate sites might be achieved by selecting the hand mixing method (see methods). The frequency of misannealing artifacts decreases towards the ligation site. (**E**) Distribution of the number of mismatches between two random UIDs when only the last six barcode positions preceding the ligation site are considered. This data was collected from the three library replicates built from patient NB-pt01 plasma. The X axis shows the number of mismatches or mispairings observed between two given barcode sequences (barcodes attached to single strands originating from independent DNA molecules in A and E and barcodes attached to each of the two parental strands of double stranded DNA molecules in (**B** and **C**). The Y axis shows the percentage of comparisons with that number of mismatches or base mispairings.

Theoretically, our semi-degenerate barcode allows for 2^24^ = 16,777,216 unique identifiers (UIDs) (2^12^ = 4,096 distinct UIDs per adapter). All PCR-derived duplicates showing a maximum of 1% mismatches, maximum gap size of 1 bp (including both the molecular tag and insert DNA sequence) and minimum overlap of 90 bp were grouped within the same family and used for the generation of single-strand consensus sequences (Minimum number of reads to generate a consensus sequence N = 3, see methods). Given the fact that the plus and minus strand of any given DNA molecule carry different tri-nucleotide tags, consensus sequences from each of the two parental strands were created independently. Thus, we identified a total of 8,283 and 37,746 UIDs from OSS-Pt01 and OVC-Pt01 (V2) libraries, respectively, associated with the consensus sequence of on-target reads mapping to the plus (5′-3′) strand (see methods). The total number of on-target reads was 450 × 10^3^ for OSS-Pt01 and 1,400 × 10^3^ for OVC-Pt01 (V2). The collapsed set of on-target reads was comprised of a mixture of consensus sequences from different parental strands, and only for a subset of molecules the two parental strands of a given cfDNA molecule were represented in the final data set. We specifically restricted our analysis of ssDNA barcode diversity to one single strand orientation to avoid any confounding issues associated with the mispairing artifacts that may have arisen during adapter annealing. The difference in the number of UIDs retrieved can be explained by both higher sequencing depth and cfDNA input for the ovarian cancer patient. Both libraries were built from the same volume (25 μl) of a cfDNA extract obtained from 2 ml plasma. Notably, ctDNA levels were remarkably higher in the ovarian cancer patient (>25%) than in the osteosarcoma patient (around 0.1%, Table 3) and we have observed that high ctDNA levels typically contribute to comparably high cfDNA yields (authors’ unpublished data). More than 99.7% of the UIDs in each library were unique and no UIDs were observed more than twice in a single data set. In practical situations, such UID clashes between molecules can still generally be distinguished as their mapping coordinates are typically distinct. The average number of nucleotide differences between all possible pairs of UIDs across the three NB-pt01 library replicates (N = 3,349), using this data set for simplicity in the calculations, was 11.39 ± 3.45 S.D. (Fig. 2, Panel A). This value is consistent with a random distribution of UIDs and suggests that each 24-nucleotide UID differs from each other (on average) in almost half of the nucleotide positions.

**Table 3:** Tracking ctDNA in the plasma of several cancer patients using semi-degenerate barcoded adapters and personalized panels of biotinylated baits.

Patient ID	Mutant ssDNA	ds Support?	ctDNA Loci	Av. ssDNA Cov	VAF Range (ssDNA)
NB-pt01-R1	180	>40 molecules	5/5	426.2	0.029–0.543
NB-pt01-R2	211	>50 molecules	5/5	501.6	0.059–0.559
NB-pt01-R3	216	>50 molecules	5/5	571.8	0.027–0.583
NB-pt02	377	22 molecules	1/1	998	0.378
NB-pt03	138 (T)	0 molecules	3/5	67/1,097	0.107–0.202
NB-pt04	>1,000	>100 molecules	4/5	1300	0.20–0.309
OVC-pt01 (V1)	2	0 molecules	1/6	2544	0.0006
OVC-pt01 (V2)	14928	>2,000 molecules	4/6	13192	0.255–0.298
OVC-pt02	215	>50 molecules	5/5	2300	0.009–0.025
OSS-pt01	11	4 molecules	3/4	4839	0.0005–0.001
OSS-pt02*	0	0	0/8	1660	0
NMC-pt01	1 (T)	0	1/1	786	0.0013
CPG-pt01*	0	0	0/2	670	0
PIB-pt01	5	2 molecules	1/1	6843	0.0007
IFB-pt01	40	9 molecules	1/1	6842	0.0058
ASL-pt01	17	1 molecule	4/5	833	0.001–0.008
SAR-pt01	2 (T)	0 molecules	1/1	635	0.0016
MGC-pt01	6	2 molecules	1/1	5384	0.001
DLBCL-pt01	260	>30 molecules	4/5	334	0.223–0.290

This table shows the total number of consensus sequences derived from one single strand that supports the presence of ctDNA at targeted loci, whether a ctDNA call has or not duplex sequencing support and the fraction of targeted sites where ctDNA has been detected. The last two columns show the average number of ssDNA consensus sequences spanning each one of the targeted sites and the range of variant allele frequencies (VAF) detected in plasma. We mostly targeted SNVs and small indels but also genomic translocations (indicated by a “T”). Patient NB-pt03 displays two values at the Av. ssDNA Cov column because two independent experiments were carried out to target SNVs and a gene fusion in the plasma.

**Figure 3:**
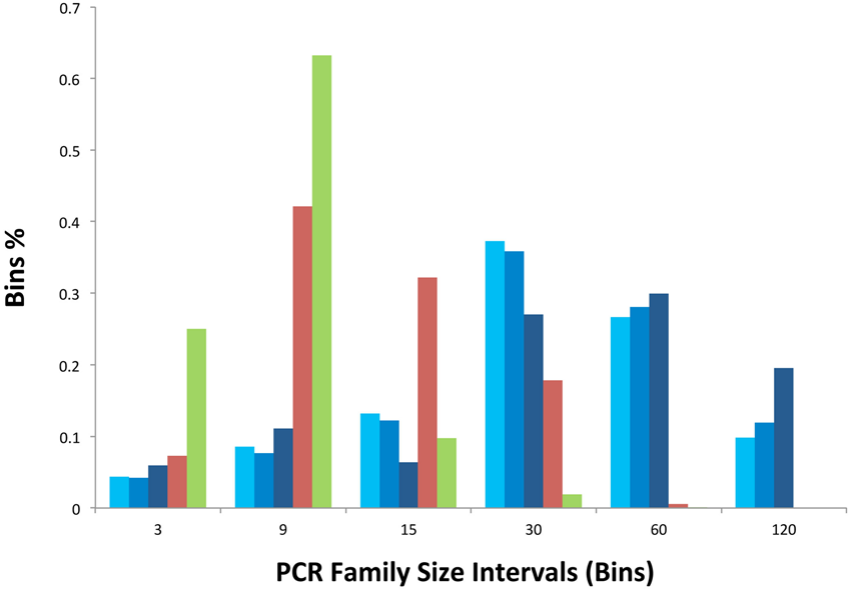
Histogram of PCR family size distribution for five cfDNA libraries built with 12-nucleotide semi-degenerate barcode adapters. The three library replicates for NB-pt01 plasma (R1, R2 and R3) are depicted by different tones of blue. The libraries built from OSS-pt01 and OVC-pt01 (V2) plasmas are indicated by red and green colors, respectively. The X axis reflects different PCR family size categories or bins and the Y axis shows the proportion of each of these categories in a given library. Singletons and consensus sequences generated from just two PCR duplicates are not included in this plot.

The PCR family size distributions for the libraries built from NB-pt01, OSS-pt01 and OVC-pt01 (V2) plasmas are summarized in Fig. 3. As expected, the diversity of the cfDNA library (estimated as the total number of unique molecules spanning targeted sites) and the number of on-target reads (estimated as the total number of reads spanning targeted sites) had a significant impact on the distribution of PCR family sizes. Thus, the three library replicates built from NB-pt01 plasma exhibited comparably larger PCR family sizes as the result of a higher ratio between the total number of on-target reads and the total number of unique DNA molecules (Tables 1 and 3). Amplification and hybridization efficiencies of each individual cfDNA fragment may explain overall differences concerning the size for each PCR family. A previous study has carefully inspected the effect of “peak” PCR family sizes, defined as the PCR family size >1 containing the highest proportion of reads^23^, on the quality of sequencing data subjected to error-suppression methods. We agree in indicating that a peak PCR family size between 6 and 12 may generate a large enough number of consensus sequences that will only slowly increase by collecting more sequencing data. We also found that the proportion of PCR families with unique mapping coordinates was as low as 55.75% in OVC-pt01 (V2) and 73.79% in OSS-pt01. Because this analysis was restricted to reads mapped to only the plus strand, these distinct families are not explained by the complement of cfDNA molecules. Based on this result, in our cfDNA samples up to ~45% of molecules shared identical genomic coordinates with at least one additional molecule. In fact, we observed as many as 63 molecules showing distinct UIDs (average number of mismatches between UIDs = 11.96 ± 2.51 S.D.) but sharing the same mapping coordinates in OVC-pt01 (V2) (see Figure S3). Likewise, Figure S4 shows a dozen of molecules carrying distinct UIDs but sharing the same mapping coordinates in OSS-pt01. We observed an increase in mapping coordinate collisions towards the center of probes, suggesting that those library fragments overlapping the most with the biotinylated probes are more likely to be retrieved due to increased hybridization efficiency. Given the nonrandom nature of fragment diversity observed here, this strongly supports the use of molecular barcoding and consensus sequencing approaches for this application. As expected, the low diversity library triplicates built from NB-pt01 plasma (see Table 3) showed, on the other hand, a much lower extent of mapping coordinates collision (88.0%, 90.8% and 92.9% unique mapping coordinates).

### Molecular matching of double-stranded (dsDNA) molecules and duplex sequencing

Because strand-specific changes resulting from polymerase errors or DNA damage can be removed, precise mutant allele calls can be derived by considering both strands, thereby suppressing artifacts other than sequencing errors or PCR misincorporations occurring late during the amplification cycle^21–23^. We confirmed that our adapters ligate efficiently to dsDNA despite the intentional mispairings in the strand-specific tag and a small number of incidental mispairings within the semi-degenerate barcode sequence (Fig. 1 and Supplemental File 1). We next characterized and quantified these misannealing artifacts as they could confound the computational matching of complementary strands of a duplex. The number of non-complementary bases between the two strands of a duplex in the low-diversity library triplicates of NB-pt01 showed a skewed distribution towards few annealing artifacts, indicating that adapter duplexes with more mispairings either do not anneal in our stringent buffer (see methods) or are not competent for ligation (Fig. 2, Panel B; see Supplemental File 1). Interestingly, we observed that annealing artifacts predominantly affected the first six nucleotides of each adapter barcode relative to the ligation site (Fig. 2, Panel D). Using this knowledge, we leverage the full length of the barcode for single strand barcoding (i.e. 24 semi-degenerate sites) but exclusively use the terminal six nucleotides preceding the ligation site (i.e. 6 + 6 = 12 semi-degenerate sites) for identifying complementary strands from the same duplex. Notably, we found that 84.2% of 1,564 reconstructed duplexes in NB-pt01 (see methods) showed three or less annealing mispairings (97.5% had five or less) across the 24-nucleotide semi-degenerate barcode. Moreover, 80% of the inferred duplexes showed zero or just one annealing artifact (17%) across the pair of 6 semi-degenerate sites flanking the ligation sites (Fig. 2, Panel C). Thus, we suggest that two given strands sharing the same mapping coordinates can be traced back to the same parental double-stranded cfDNA molecule if they show alternative orientations/strand-specific tags and do not differ in more than two mismatches across this region. In order to match the two strands of a given cfDNA molecule, this 12-nucleotide semi-degenerate barcode allows for 2^12^ = 4,096 UIDs. We evaluated the diversity of this region across the most diverse cfDNA library in our data set (OVC-pt01 (V2); Table 3). We retrieved 3,979 12-nucleotide UIDs (97.49% of possible barcodes) in a sampling of 37,746 single stranded consensus sequences with distinct 24-nucleotide UIDs. The most commonly observed 12-nucleotide UID was found 52 times, which suggests a maximum probability for perfect barcode overlap of 0.013 across this region. For two molecules to be incorrectly matched as a duplex they would need to share 10 out of the 12 nucleotide positions comprising the duplex-specific UID and mapping coordinates. The average number of mismatches between two random 12-nucleotide UIDs was 6.01 ± 1.72, indicating that annealing artifacts, sequencing errors and PCR errors are unlikely to generate clashing UIDs (Fig. 2, Panel E). Longer regions of the barcode can be used to resolve infrequent duplex UID clashes if annealing mispairings are lacking or reduced in a way that molecule discrimination can be achieved unambiguously.

Previous studies have shown that recovery rates of reads representing both strands of a duplex can be disappointedly low for large genomic targets^21, 23^. Here, we report recovery rates for duplexes close to 50% in some of our enrichment experiments relying on reduced sets of personalized baits. We observed that duplex recovery efficiencies slightly increased with the amount of on-target sequence data (Table 1) in the three NB-pt01 replicates (0.36, 0.37 and 0.41 for R1, R2 and R3, respectively). As clearly shown in a previous study, duplex sequencing recovery rates may only slightly increase with deeper sequencing after an optimal “peak” PCR family size^23^. We observed that library diversity and the size of the personalized panel of biotinylated baits were also important, as exemplified here by the lower duplex sequencing recovery efficiencies observed in patient OVC-pt01 (V2) (0.16) compared to patient OSS-pt01 (0.47). Importantly, we also found a strong concordance between the VAFs inferred through the exclusive analysis of ssDNA consensus sequences and the VAFs calculated through the exclusive analysis of molecules with duplex support (VAF range = 0.027–0.583 versus 0.029–0.611 in the three NB-pt01 replicates; VAF range = 0.255–0.298 versus 0.239–0.285 in patient OVC-pt01 (V2); VAF range = 0.0005–0.001 versus 0.001 in patient OSS-pt01). Even though restricting the analysis to cfDNA fragments with duplex support decreases false positive detection rates (see next section) this strategy also inevitably decreases the number of unique molecules spanning each targeting site, and it therefore may negatively impact assay sensitivity. It also remains unclear why a substantial fraction of library fragments appear to generate reads from only one half of the duplex and methodological enhancements to improve this are another important area of future exploration.

### Assessment of error-suppression

**Figure 4:**
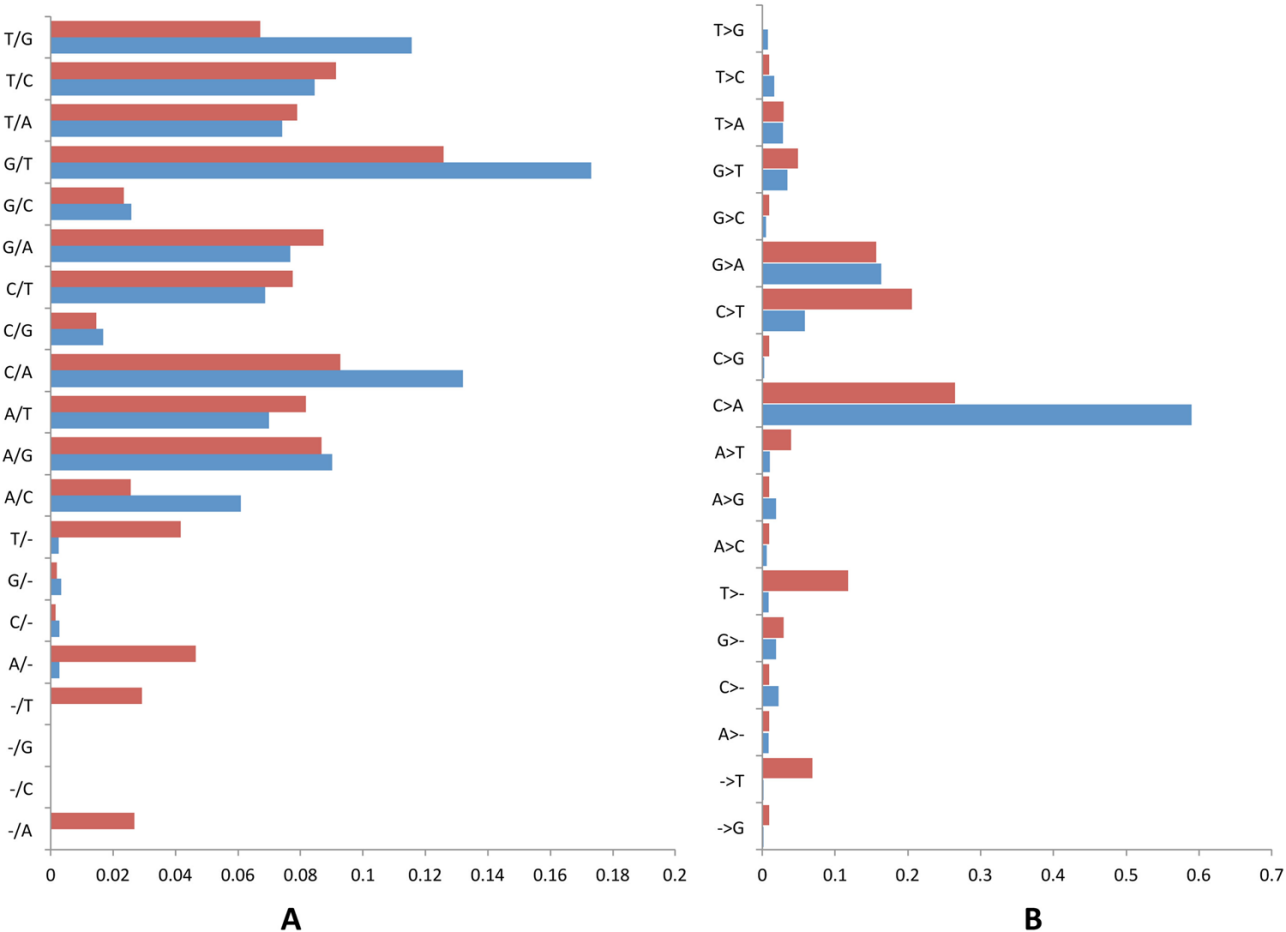
Landscape of background errors in two diverse cfDNA libraries (OVC-pt01 (V2), blue bars; OSS-pt01, red bars). Panels A and B list the type of errors that are corrected at the ssDNA or dsDNA consensus phases, respectively. The Y axis shows different types of presumably false variants and the X axis shows the observed frequency for each of these putative artifacts.

We observed that single strand consensus sequencing corrected indels and all 12 substitution classes in OSS-pt01 and OVC-pt01 (V2) libraries (Fig. 4, Panel A). Slight dissimilarities between libraries could be attributed to variable sequence context within the small genomic regions targeted (e.g. homoadenine and homothymine related-indels in OSS-pt01). To quantify the utility of single and duplex-consensus sequences in reducing the error rate, we investigated the fraction of non-reference allele calls (excluding *a priori* known SNPs and somatic mutations) out of the total number of bases investigated. Overall, ssDNA consensus sequencing diminished background noise down to 3.67 × 10^−5^ and 7.29 × 10^−5^ in OSS-pt01 and OVC-pt01 (V2) libraries, respectively. We observed that stereotypical errors like C > A transversions, and to a lesser extent, G > A and C > T transitions, were highly resilient to single-strand consensus sequencing (Fig. 4, Panel B; see also Figure S5). We also observed a strong imbalance of C > A versus reciprocal G > T non-reference allele calls in the two libraries investigated that could not be explained by strand bias during sequencing. DNA damage occurring before library preparation is expected to generate a balanced ratio of G > T to C > A artifacts, as damage may occur randomly in any of the two strands. The pattern observed in this study is therefore consistent with oxidative DNA damage leading to 8-oxoguanine and cytosine deamination during the hybridization of library-derived minus strands and our biotinylated plus strands (see methods; Figure S6; see also ref. 21). We have observed a consistent and high predominance of C > A false positives after ssDNA consensus in all cfDNA libraries we have worked with up to the date, pointing out that this evidence of DNA damage was not just limited to the two libraries analyzed in detail here. The two strands of the initial cfDNA fragments can be distinguished through their mapping orientation, strand-specific tags and UIDs. As such, errors not represented on reads from the complementary strand of the duplex can be ignored. Using this approach, background noise was further decreased down to 3.30 × 10^−6^ in OSS-pt01 and 5.60 × 10^−7^ in OVC-pt01 (V2).

### Sensitive detection of ctDNA in the plasma of cancer patients using personalized baits

We first evaluated the performance of our hybridization capture-based method on serial dilutions of a cell line (DB) genomic DNA carrying a mutation (*EZH2* p.Y641N) that was covered by our lymphoma-specific capture gene panel. The genomic DNA of this cell line DNA was mechanically sheared to an average of 200 bp using an ultra-focused sonicator to mimic the size distribution of cfDNA. Inferred allele frequencies for mutant DNA fitted expectations (Table S2). We also investigated the influence of genomic DNA contamination when inferring ratios of mutant versus wild-type DNA. Genomic DNA shed into blood samples as the result of cell lysis can result in the overestimation of circulating wild-type alleles. Long DNA molecules, however, did not make into library fragments and therefore we did not observed more diluted mutant DNA signals as a result of high molecular weight DNA contamination. This finding could be considered an advantage of ligation-based approaches over PCR-based methods (Table S2).

After this preliminary validation, we performed personalized ctDNA assays on 14 additional cancer patients diagnosed with a broad spectrum of malignancies (Table 1) using between one and six biotinylated DNA baits per patient (Table 3). We successfully quantified ctDNA with the aid of our semi-degenerate barcoded adapters and user-friendly bioinformatics workflow (see methods) in all but two of the cases investigated. Basically, we generated consensus sequences from at least three PCR copies of each parental strand of any given double stranded DNA molecule by leveraging the single-molecule tagging information associated with our molecular barcodes. Estimated variant allele frequencies (VAFs) for each of the somatic mutations targeted in the present study together with some descriptive statistics are provided in Tables 1 and 3. The genomic coordinates of each somatic mutation detected in the plasma of these patients are listed in Table S1. The two cases (OSS-pt02 and CPG-pt01) negative for ctDNA were confirmed as such by droplet digital PCR (ddPCR). Patient OSS-pt02 was diagnosed with metastatic osteosarcoma, with an attempt to surgically resect the two pulmonary nodules revealed by a CT scan. Patient CPG-pt01 was diagnosed with progressive adamantinomatous craniopharyngioma, treated surgically, with no metastatic dissemination. Several assays failed to detect any mutant-positive droplet in either OSS-pt02 or CPG-pt01 plasma samples in spite of the counting of >35,000 and >2,700 wildtype DNA molecules, respectively. The lower limit of detection for our targeted hybridization capture experiments was at least 0.1%. As many as three different plasma samples (collected at different time points during the course of the disease) were investigated in patient CPG-pt01 and all were deemed negative for ctDNA.

All but one of the positive cases (OVC-pt01 (V1)) in which we targeted somatic SNVs exhibited duplex sequencing support (Table 3). The list of positive cases for ctDNA with duplex support included two additional neuroblastoma patients (NB-pt03 diagnosed with stage IV neuroblastoma), one patient diagnosed with ovarian cancer (OVC-pt02), one patient diagnosed with infantile fibrosarcoma (IFB-pt01), one patient diagnosed with hepatic angiosarcoma (ASL-pt01), one patient diagnosed with metastatic granular cell tumour (MGC-pt01) and one patient diagnosed with metastatic diffuse large B-cell lymphoma (DLBCL-pt01). We allowed for a maximum of 2 UID mismatches between strands for those libraries built with five-nucleotide semi-degenerate barcoded adapters (see methods). Duplex support was not critical for two cases targeting genomic rearrangements, as these variant calls are highly specific. We found two molecules supporting an *EWSR1-ATF1* gene fusion in a pediatric sarcoma patient (SAR-pt01, VAF = 0.0016) showing metastatic lesions in the lung and one molecule supporting a *NUTM1-BRD*4 gene fusion in a pediatric patient diagnosed with stage IV NUT midline carcinoma and widespread metastatic lesions (NMC-pt01, VAF = 0.0013) (Tables 1 and 3). A third genomic rearrangement (*ALK-NEURL1*) was detected in the plasma of a stage IV neuroblastoma patient (NB-pt03) and further confirmed by digital PCR, yielding similar VAFs (0.109 versus 0.069, respectively). All cases were consistent with the breakpoint observed in these patients’ tumor genomes.

The lowest ctDNA levels were detected in patients OSS-pt01 and PIB-pt01, diagnosed with metastatic osteosarcoma (pulmonary lesions) and pineoblastoma, respectively. We found tumor-derived DNA in the plasma of OSS-pt01 at allele frequencies ranging between 0.0005 and 0.001. We retrieved 11 mutant ssDNA molecules from which 8 could be reconstructed into 4 duplexes. At least one duplex, matching the mutations previously identified in the tumor biopsy, covered each of the three sites positive for ctDNA. Similarly, we detected a somatic *TP5*3 mutation with an allele frequency of 0.00087, supported by two independent DNA duplexes, in the plasma of the brain cancer patient. Low ctDNA levels in this patient were not surprising because of the well-known effect of the blood-brain barrier^12, 13^.

### Sequencing cfDNA libraries with gene panels and measuring ctDNA dynamics

We assessed a commercially available panel covering 128 cancer-related genes (XGen**®** Pan-Cancer Panel, Integrated DNA Technologies) and a separate disease-focused panel of 72 lymphoma-related genes that was specifically generated in our laboratory from individual XGen**®** Lockdown probes (see methods). For example, we detected high levels of ctDNA in an acute lymphoblastic leukemia patient (ALL-pt01) using the XGen**©** Pan-Cancer Panel. Two somatic variants at *KIT* and *SMC3* genes were found with high VAFs in the plasma of this patient (VAFs = 0.436 and 0.358; 661x and 81x coverage, respectively; see Table S1 for genomic coordinates). Notably, we also detected an activating *KRAS* p.G12A mutation in the plasma of this patient but at a much lower VAF (0.03, 198x coverage). The three variants matched somatic mutations previously identified in the primary tumor, where there was strong evidence to classify the *KRAS* mutation as subclonal as well. In a different example, a somatic *TP53* nonsense mutation with a high VAF = 0.608, was reported in the plasma of a stage IV Ewing sarcoma patient (ESR-pt01, 199x coverage; Table S1) after leveraging the XGen© Pan-Cancer Panel. This somatic mutation was previously found in the tumor DNA, together with evidence supporting loss of heterozygosity at the *TP53* locus. The normal DNA of this patient, on the contrary, was homozygous for the wild-type allele. We also identified as many as 9 variants in the plasma of a Hodgkin lymphoma patient (HGL-pt01) that were not previously identified in the tumor biopsy (Table S1) after combining the XGen Pan-Cancer Panel and our specific lymphoma-related gene panel. These variants were supported by a minimum of three consensus sequences derived from single strands and independent molecules and were not related to the persistent C > A false positives. Tumor whole genome sequencing is particularly challenging in Hodgkin lymphoma as the involved disease sites predominantly consist of reactive cell populations with scarce, malignant Reed Sternberg cells present. A *TBL1XR1* splice site mutation (VAF = 0.083, 557x coverage) showed the highest support for ctDNA, including a total of 44 consensus sequences derived from one of the parental strands and one molecule with duplex support. Among the mutations we also noted a *TP53* missense mutation with support from 10 ssDNA molecules (see Table S1).

**Figure 5:**
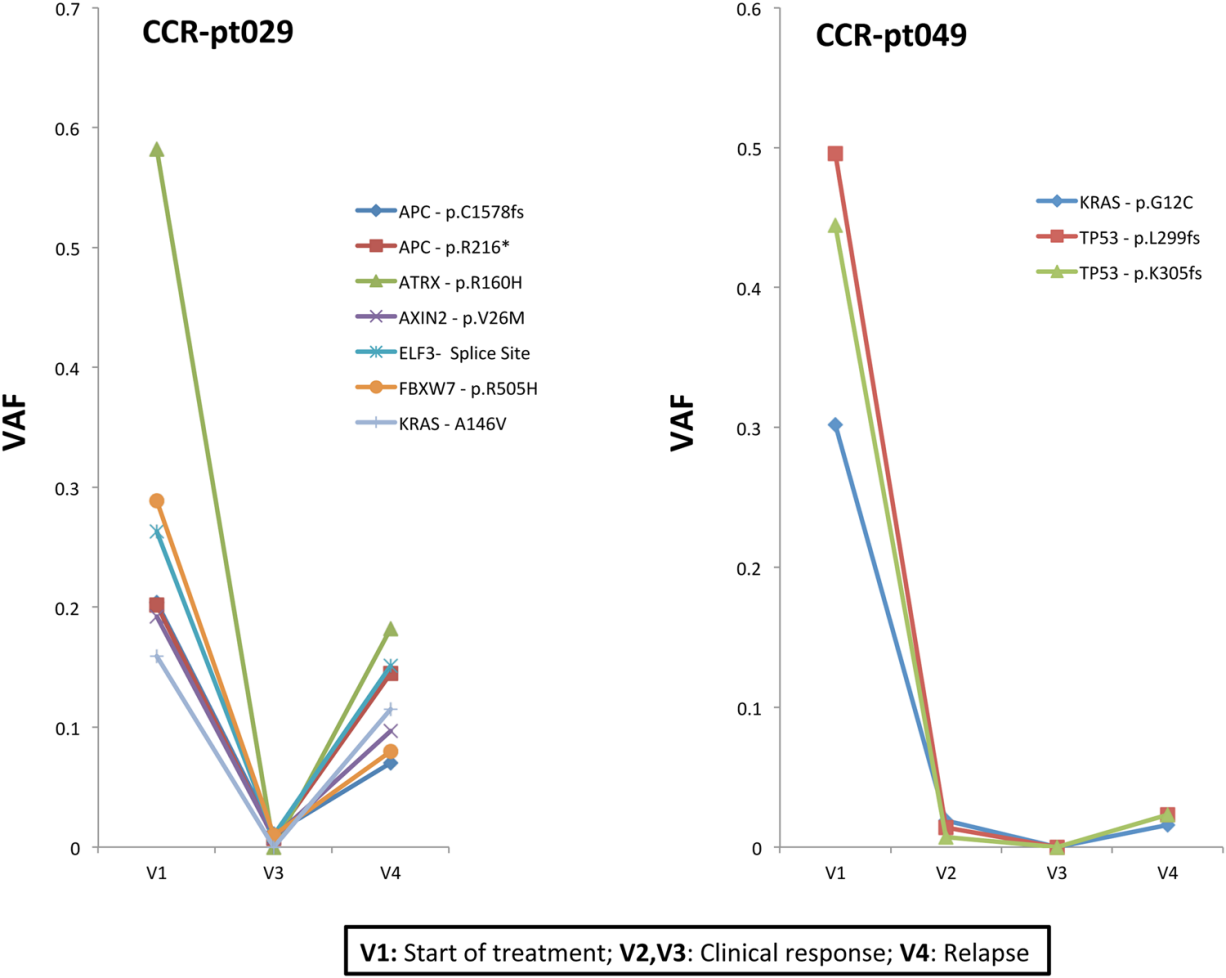
Monitoring of ctDNA abundance across longitudinal plasma series drawn from two colorectal cancer patients (CCR-pt029 and CCR-pt049). V1 relates to the plasma sample drawn at the beginning of the therapeutic treatment. Plasma samples collected at V2 and V3 time points show a decrease in ctDNA levels, in agreement with clinical response. The two patients relapsed after several weeks and this fact is consistent with the raise of ctDNA levels observed in the plasma sample collected at V4 time point. The Y axis indicates the variant allele frequency (VAF) of the somatic mutations quantified in plasma. These libraries were constructed with 12-nucletoide semi-degenerate barcoded adapters and enriched with a panel targeting the exons of 128 cancer-related genes.

The XGen**®** Pan-Cancer Panel was also applied to series of plasma samples drawn from two patients with metastatic colorectal cancer (CCR-pt029 and CCR-pt049) to assess its potential for patient monitoring. We found large fluctuations in ctDNA abundance, using somatic SNVs and indels as reporters, among the plasma samples analyzed (Fig. 5) that were in general agreement with the progression of the disease. In both patients, ctDNA levels were high before the initiation of the therapeutic treatment, (V1) decreased over the period of clinical response (V2 and V3) and then increased again after relapse (diagnosed by surveillance CT scans, V4). Notably, we also found a strong correlation between the VAFs inferred through our targeted hybridization capture and the VAFs inferred through an amplicon sequencing experiment previously conducted for the same set of samples in our lab (r^2^ = 0.91, Figure S7, see methods). We also had a series of plasma samples from a lymphoma patient enrolled on a recently completed clinical trial that were enriched with our set of lymphoma-related capture baits. All four samples preceding this patient’s ultimate disease progression showed no indication of ctDNA. The sample collected at progression (206 weeks) showed evidence of mutations in both *FOXO1* (p.M1V, p.I10V) and *BCL2* (p.P59A) with an average VAF of 0.025. Other mutations in this patient’s initial tumor biopsy were not detected in the sequence data. However, using a recently developed ddPCR assay^24^ we detected ctDNA in *STAT6* (p.D419G) with a VAF of 0.003 from the same sample collected at the time of relapse. Both hybridization capture and ddPCR detected ctDNA from only this sample, suggesting that minimal residual disease was present but not detected in earlier samples. Notably, the plasma volumes used for these assays (~0.5 mL) were significantly lower than desirable for detecting ctDNA levels much below 1%.

Finally, we also had access to a series of plasma samples (N = 6) from two breast cancer patients (Neo-02 and Neo-027), five from early stages of the disease and one collected at the time of metastasis. We enriched these plasma-derived DNA libraries with baits exclusively targeting the entire coding region of *TP53* based on *a priori* information derived from tumor biopsies. We detected duplex-supported ctDNA in four of these cases, with VAFs ranging from 0.011 to 0.048 (see Table S1 for the genomic coordinates of mutations). Importantly, we observed a strong correlation between the VAFs inferred by our capture-based sequencing method and ddPCR (r^2^ = 0.94, Figure S7) in the same set of samples. The ovarian granulosa patient (OVC-pt01) represents another case where at least two plasma samples were collected at different time points. We report only two ssDNA molecules across the personalized panel in a blood sample (V1) that was drawn 9 months prior to a second sample (V2) in which we detected ctDNA at VAFs higher than 20%. This finding is therefore a strong indicator of disease progression in this patient.

## Discussion

Liquid biopsies offer numerous potential avenues to advance personalized medicine including noninvasive genotyping, early assessment of therapeutic response, timely detection of treatment-resistant mutations and recognition of minimal residual disease in cancer^1–16^. Accurate detection of ctDNA, however, needs to be supported by molecular approaches with ultimate sensitivity and specificity^17–19, 21–23, 25, 26^. One of the major advantages of our error-suppression sequencing method is the cost-effective production of barcoded adapters that are compatible with standard ligation-based library preparation methods. Our method to generate double-stranded adapters does not require additional enzymatic reactions or multiple complex hybridizations while still allowing for recovery efficiencies of cfDNA fragments equivalent to those reported by previous approaches^7, 21, 23^. Although barcode mismatches between the two strands of a duplex due to misannealing are a potential shortcoming of our strategy, we have demonstrated that these situations involve a small number of bases per duplex and can thus be easily recognized and handled in the resulting data. Another benefit of our adapter design includes the large diversity of our barcode, which allows for recognition of large pools of molecules sharing the same coordinates. This phenomenon is significantly more relevant as assays are designed to target restricted genomic spaces and yield a concomitant greater sequencing depth. Importantly, the extra length of the DNA insert is unlikely to impact our ability to read entire cfDNA fragments through standard 125 or 150 paired-end sequencing given their average size (~ 170 bp)^16^. Recent studies have also highlighted that ctDNA is usually more fragmented than somatic DNA^27, 28^. Thus, ctDNA might be better represented in small cfDNA fragments and sequencing a slightly larger DNA insert will not severely compromise sensitivity.

All ctDNA assays are subject to potential sources of error including cross-contamination and artifacts, such as those originating from DNA damage, which can resist error correction^21^. We believe that the landscape of background errors shown in this and previous studies^21^ may guide the selection of personalized biomarkers to track ctDNA levels, when possible. For example, somatic mutations involving G > C or C > G changes must be preferred over C > A or G > T changes, as the former are definitively associated with lower false positive rates. Structural variants such as those targeted in the present study and others^29, 30^, on the other hand, may yield very specific assays and require comparably lower amounts of sequencing data (see for instance NMC-pt01, Tables 1 and 3). Given the accuracy of error-corrected methods, assays relying on targeted hybridization capture may not only be useful to noninvasively monitor disease progression by means of personalized panels but may also benefit from the inclusion of panels targeting *de-novo* resistance-acquired mutations or other mutations with prognostic value. For example, simultaneous surveillance of *KRAS* and *EGFR* hotspot mutations in colorectal and lung cancer^10, 18, 31^, *STAT6* hotspot mutations in diffuse large B-cell lymphoma^32^ or *ABL1* mutations in chronic myeloid leukemia^33^, may afford opportunity to recognize acquired treatment resistance prior to clinical signs of relapse.

A single capture bait may enable detection of any mutations in the captured region, including less common variants that would be otherwise missed by digital PCR assays targeting the most commonly observed mutations^24, 34^. Moreover, relative differences in clonal prevalence of individual mutations are typically not observable in dPCR due to its limited multiplexing capabilities^24, 34–36^. The lack of any error-suppression method is another factor that may comparably compromise the specificity of dPCR under certain scenarios^37, 38^ or when pre-amplification steps are performed. Personalized experiments relying on biotinylated DNA baits may also imply lower costs, as acquiring more than three hydrolysis probe-based assays for digital PCR can be very expensive and such assays may also need optimization. For cases where well-established hydrolysis probe-based approaches are available (i.e. digital PCR assays targeting recurrent mutation hotspots in certain cancer types^24, 34^), turnaround times can be longer when applying hybridization capture approaches, particularly when two rounds of enrichment are required to achieve desired fractions of on-target reads^39^.

We also explored sequential hybridizations in an attempt to increase the proportion of reads representing the target loci during personalized assays. Since the fraction of on-target reads barely reached 5% after applying one single round of targeted enrichment with small panels of personalized baits, two successive rounds of hybridization were strictly necessary for those cases with low levels of ctDNA. We did not observe, however, the high (>90%) on-target efficiencies reported by early reports of that strategy^39^, possibly relating to the size of our personalized panels, which were at least ~5-fold smaller (<1 kb) than that used in the cited study. Our enrichment efficiencies after two rounds of capture were >85%, nevertheless, when using a pool of probes spanning the entire coding region of *TP53*. Adding a third round of capture may elevate the fraction of on-target reads during personalized assays^39^ but this remains to be explored. In any case, the fraction of on-target reads is a parameter that can greatly fluctuate depending on the specific pools of biotinylated probes employed, being the number of biotinylated probes, their distribution and their sequence context and complexity important factors. For example, we observed around 40% on-target reads when leveraging the XGen**®** Pan-Cancer Panel but around 80% on-target reads when using our lymphoma-specific gene panel. In both cases, a single round of hybridization captures was carried out.

Unlike other approaches, our adapter design contains a region that can be customized to produce distinct sample-specific tags in individual libraries beyond those regions typically employed for library indexing purposes. This provides an inherent means to recognize and ameliorate sources of cross-contamination between experiments, including the undesired effect of index “cross-talk” during sequencing. Strand-specific tags can also be altered to increase nucleotide diversity during sequencing, as low sequence diversity may cause low numbers of clusters passing filter in Illumina sequencers. For example, we have intentionally altered the semidegenerate barcode composition across new sets of adapter constructs and detected no negative effects regarding the incidence and frequency of annealing artifacts in DNA duplexes (authors’ unpublished data). Our approach also allows incorporating more than one type of adapter construct to tag the same single library if extreme molecule discrimination is intended, then adding great flexibility to this technology.

In summary, we have demonstrated the application of our method to a broad range of target regions and disease conditions. Selecting assays that balance library diversity, target space and ctDNA abundance can help maintain lower costs as these approaches gain adoption for research and clinical applications. Opting for high depth across small-targeted spaces to maximize the number of cfDNA molecules represented by the two parental strands, in cases expected to have low ctDNA, and broader gene coverage in patients with cancers that tend to have higher ctDNA (where duplex sequencing support is not that critical) is recommended. It must be noted that the incorporation of our barcoded adapters only involves a small extra cost per library (5–6 $USD) without any evident downside regarding the use of standard sequencing adapters. In the absence of tumor sequencing data, and under scenarios with sufficiently high ctDNA levels, we also believe that broad cancer-related gene panels may assist the design of personalized panels to monitor disease progression, more cost-effectively, across longitudinal liquid biopsy samples. In conclusion, we have implemented a highly sensitive strategy for the detection and monitoring of ctDNA that offers appealing features including flexibility and accuracy, which we hope will allow for adoption in research and ultimately clinical settings.

## Methods

### Human subjects

Nineteen patients enrolled in the Personalized Oncogenomics (POG) project provided plasma samples for the present study (see Table 1). Seventeen of these were pediatric patients. The POG study represents an ongoing collaborative effort between scientists, bioinformaticians and clinicians at the BC Genome Science Centre, BC Cancer Agency and BC Children’s Hospital to molecularly characterize relapsed or refractory cancers (Laskin *et al*. 2015 Cold Spring Harb Mol Case Stud 1: a000570). Patients (≥1 year) with relapsed, refractory or very poor prognosis cancers who had either failed standard therapy approaches or who had a very low likelihood of cure were enrolled following informed consent (and assent). Whole genome and transcriptome sequencing of a tumor sample obtained at study entry, along with a matched germline sample was undertaken.

Plasma samples were collected from two colorectal cancer patients taking part of the Q-CROC-01 trial “Prospective study to identify molecular mechanisms of clinical resistance to standard first-line therapy in patients with metastatic colorectal cancer (NCT00984048)”. Patients receiving FOLFOX (leucovorin, 5-fluorouracil and oxaliplatin) and bevacizumab consented to serial collection of tumor biopsies and blood at baseline and over time of treatment. Written informed consent for research biopsies and blood sampling was also obtained from two breast cancer patients (Neo-02 and Neo-27) that had participated in the Q-CROC-3 clinical trial (NCT01276899). Finally, plasma samples were collected from a patient with diffuse large B-cell lymphoma following informed consent in the context of a recently completed clinical trial. Details of the larger patient cohort, including the case analyzed here, can be found in the paper detailing that trial^8^. Blood samples drawn from this patient ranged from 1 to 6 months apart.

### Sample acquisition

All patients provided written, informed consent. This project was approved by the research ethics boards at the Jewish General Hospital, British Columbia Cancer Agency, British Columbia Children’s Hospital, Simon Fraser University and is in accordance with the declaration of Helsinki. Approximately 30 mL of total blood was drawn from 19 POG patients and stored in 10 ml Cell-free DNA BCT^®^ tubes (Streck) during a maximum of two weeks at room temperature. Blood samples drawn from two patients diagnosed with cancer of the ovary were preserved in EDTA tubes. Approximately 15 mL of total blood from colorectal cancer patients were collected in k-EDTA tubes, centrifuged to separate plasma from peripheral blood cells within 30 min of collection, and fractions were frozen at −80 °C. Approximately 10 mL of total blood was drawn from breast cancer patients and were collected in CTAD tubes at various time points. Tubes were processed and plasma isolated within 2 h of blood draw and immediately stored at −80 °C. Cell-free DNA was isolated from 2–4 ml of plasma, when possible, using the Qiamp circulating nucleic acid kit (Qiagen) and eluted in 50 µl of AVE buffer (Qiagen).

### Adapter design

Our first adapter design consisted of annealing two oligonucleotides that were slightly modified in their 3′-end with respect to the oligonucleotides that comprise standard Y-shaped Illumina sequencing adapters. These modifications consisted of the addition of a five semi-degenerate and potentially complementary barcode tag followed by a fixed and fully complementary tri-nucleotide tag. The sequence of these two oligonucleotides are: Ad-WSWSW-Tag-01: 5′-ACACTCTTTCCCTACACGACGCTCTTCCGATCT*WSWSW* GAC*T-3′; Ad-WSWSW-Tag-02: 5′-/5Phos/**GTC** *WSWSW*AGATCGGAAGAGCACACGTCTGAACTCCAGTC-3′ (*a phosphorothioate bond; the fixed and complementary tri-nucleotide tag is indicated in bold; the semi-degenerate barcode tag is shown in italics). This design can theoretically generate 2^5^ = 32 unique tags for single adapters and 32^2^ = 1,024 unique tags for cfDNA fragments locked in between two adapters. Even though this adapter design performed relatively well, we observed that annealing artifacts caused by the imperfect pairing of the two oligonucleotides could hinder our capabilities to accurately match the two strands derived from the same cfDNA molecule.

In an effort to minimize the impact of annealing mispairing during duplex sequencing we performed two modifications. First, we extended the length of the semi-degenerate barcode tag from five to twelve nucleotides to increase barcode diversity. Second, we placed the tri-nucleotide tag immediately before the barcode tag. An important difference with respect to the previous adapter design is that tri-nucleotide tags are not complementary. The main goal of introducing mispairing in this region is to decrease annealing mispairings along the semi-complementary region. We hypothesized that oligonucleotides will be “more obliged” to a better complementary annealing in order to generate adapters that are competent for ligation. The sequences of the two oligonucleotides that compose this new adapter design are: Ad-12nt-TagPlus: 5'- ACACTCTTTCCCTACACGACGCTCTTCCGATCT**NNN** WSMRWSYWKMWW*T-3'; Ad-12nt-TagMinus: 5'- /5Phos/*WWKMWRSWYKSW* **NNN**AGATCGGAAGAGCACACGTCTGAACTCCAGTC-3'. The variable tri-nucleotide tag is in bold and the 12-nucleotide semi-degenerate tag is shown in italics (see Fig. 1 and Supplemental File 1 for more details). These two variable oligonucleotide sequences are thereafter referred as “adapter-plus” or “adapter-minus”, respectively. Adapter oligonucleotides were individually synthesized by Integrated DNA Technologies at the 100 nmol synthesis scale, selecting the machine mixing randomization method, purified by HPLC and normalized at 100 µM in TE Buffer (pH = 8.0).

### Barcoded adapter annealing

“Adapter-plus” and “adapter-minus” 12-nucleotide semi-degenerate oligonucleotides were pooled in equimolar concentrations in a final volume of 50 µl containing 2.25 nmol of “plus” oligonucleotides, 2.25 nmol of “minus” oligonucleotides and a final 1X concentration of stringent wash buffer (NGS Hybridization and Wash Kit, Integrated DNA technologies) to minimize non-complementary base pairings. “Plus” and “minus” oligonucleotides can be combined in different ways to generate adapter populations that differ in the sequence of the two strand-specific tags. For example, four “minus” and six “plus” oligonucleotides can generate as many as 19 unique and non-complementary combinations of strand-specific tags (Table S3). Mixtures of “plus” and “minus” oligonucleotides were initially denatured at 98 °C during 5 min in a thermocycler with the lid pre-heated at 105 °C. Temperature was then decreased at a rate of −0.1 °C per second until reaching 25 °C, from where the incubation was extended during 60 min at this temperature. Adapter stocks were stored at −20 °C for a maximum of six months. The annealing of our previous adapter design consisted on the mixing of 1 nmol of the Ad-WSWSW-Tag-01 and 1 nmol of the Ad-WSWSW-Tag-02 oligoes in a final volume of 100 µl containing 1x concentration of annealing buffer (100 mM Tris-HCl, pH = 7.5, 10 mM EDTA, 1 mM NaCl).

### Library preparation

Some cfDNA libraries were prepared using the KAPA LTP Library Preparation Kit for Illumina platforms (Kapa Biosystems), according to the protocol described in ref. 7, whereas others relied on the NEBNext^®^ Ultra^TM^ II DNA Library Prep Kit for Illumina (New England BioLabs). The latter method was found to be less laborious but showed overall comparable performance. The amount of cfDNA input varied by patient, mostly depending on yield, but basically we built libraries using 25 µl of the 50 µl cfDNA extracts obtained after processing 2–4 ml of plasma. During the use of the NEBNext^®^ Ultra^TM^ II DNA Library Prep Kit, DNA is end-repaired and A-tailed in a final volume of 60 µl following the manufacturer’s protocol, foregoing the fragmentation step typical of most library preparation procedures. The ligation reaction was carried out in the presence of at least 100-fold excess of barcoded adapters during 15 min at 20 °C and then incubated overnight at 16 °C. In the case of our 12-nucleotide semi-degenerate barcoded design, we added 5 µl of annealed adapters (see above) and slightly adjusted the volume of the NEBNext Ultra II Ligation Master Mix (30.5 µl instead of 30 µl per reaction). The ligation reaction was cleaned with 0.8x volumes (76 µl) of Agencourt AMPure® XP magnetic beads (Beckman Coulter). Two 75% ethanol washes were performed in a way that beads were fully resuspended in the ethanol. This strategy has been suggested to improve the removal of adapter dimers^23^. Ligated cfDNA products were eluted in 25 µl of 10 mM Tris-HCl pH = 8.0 and mixed with 5 µl of indexed PCR primers at 10 µM each and 30 µl of NEBNext Ultra II Q5 Master Mix (New England BioLabs). The sequences of our PCR primers, suitable for dual-indexing in Illumina platforms, are: 5′-CAAGCAGAAGACGGCATACGAGAT**NNNNNN**GTGACTGGAGTT3′ and 5′-AATGATACGGCGACCACCGAGATCTACAC**NNNNNN**ACACTCTTTCCCTACACGACGCTCTTCCGATC*T-3′. The position of the variable 6-nucleotide index is indicated in bold. The PCR protocol consisted of an initial denaturalization step at 98 °C during 30 sec followed by 8–9 cycles of 98 °C during 10 s and 65 °C during 1:30 min. There was a final incubation step at 65 °C during 5 min before keeping the amplified libraries at 4 °C indefinitely. Libraries were then purified using 0.8× volumes (48 µl) of Agencourt AMPure XP beads ensuring again that beads were fully resuspended in 75% ethanol during the washes. Final library were eluted in 25–30 µl of 10 mM Tris-HCl pH = 8.0 and total yields were measured in a Qubit fluorometer. Some libraries were verified using an Agilent Bioanalyzer instrument (Agilent Technologies) and subjected to a second round of clean-up using 0.8× volumes of AMPure XP beads if substantial amounts of adapter dimers were detected. This additional clean-up step is usually necessary when building libraries from small cfDNA inputs (e.g. <10 ng).

### In-house generation of DNA biotinylated baits

We designed four pairs of PCR primers to amplify short DNA fragments (~120 bp) containing somatic SNVs in patient OSS-pt01. These primers contained a locus-specific sequence to amplify the gene of interest plus M13 tails at their 5′ end. Primers were synthesized by Integrated DNA Technologies and purified using standard desalting conditions. The four loci were simultaneously amplified in one single 10 µl PCR reaction using 5 µl of the Kapa HiFi HotStart DNA polymerase ReadyMix (Kapa Biosystems), 0.2 pmoles of each primer and about 3 ng of wild-type human genomic DNA (Promega). The cycling protocol consisted of an initial step at 95 °C during 3 min followed by 15 cycles of 98 °C during 15 sec, 58 °C during 30 sec and 2 min extension times at 72 °C; there was a final incubation step at 72 °C during 4 min. PCR amplicons were subjected to a double size-selection using Agencourt AMPure XP beads. First, we added 0.5X volumes (5 µl of magnetic beads) to remove the genomic DNA that served as template for our first round of amplification. The PCR amplicons that were not bound to the beads were then purified with 1.5X volumes of magnetic beads and eluted in 20 µl of 10 mM Tris-HCl pH = 8.0. The second round of amplification was carried out in a final volume of 50 µl containing 20 µl of eluted amplicons, 20 pmol of a 5′ biotinylated universal primer: 5′Biotin-GTTTTCCCAGTCACGAC-3′, 20 pmol of a 5′-phosporylated universal primer: 5′P-CAGGAAACAGCTATGAC-3′ and a final 1x concentration of fresh Kapa HiFi HotStart DNA polymerase ReadyMix. Universal 5′Biotin and 5′Phosphorylated primers were synthesized and HPLC-purified by Integrated DNA Technologies and were complementary to the M13 tails included in the primers used during the first round of amplification. We performed 35 additional cycles but at an annealing temperature of 60 °C. The new batch of PCR amplicons was purified with 1.5× volumes of Agencourt AMPure XP magnetic beads, eluted in a final volume of 25 µl and quantified in a Qubit fluorometer. Aliquots of PCR products at ~15 ng/µl were now treated with 25 U of lambda exonuclease (New England Biolabs) in a final volume of 50 µl containing 1x final concentration of lambda exonuclease reaction buffer (New England Biolabs). The reaction was incubated during 30 min at 37 °C and the enzyme was heat-inactivated at 75 °C during 10 min. Single-stranded biotinylated baits were purified with 1.5x volumes of AMPure XP beads and finally eluted in 25 µl of 10 mM Tris-HCl, 0.1 mM EDTA, pH = 8.0 buffer. In a similar way, we produced biotinylated baits targeting a somatic indel in patient NB-pt02. We also designed a biotinylated amplicon to target a *NUMT1-BRD4* gene fusion in patient NMC-pt01 Our pair of primers amplified the wild-type sequence of the *NUMT1* gene flanking the fusion breakpoint, which was previously determined by whole genome sequencing of the primary tumor.

### Targeted enrichment and sequencing of cfDNA libraries

Individual or pooled libraries were mixed (at least 500 ng per pool) with 5 µg of Human Cot-1 DNA (Thermo Fisher Scientific), 1 nmol of xGen® Universal blocking oligo-TS HT-i7 and 1 nmol of a custom xGen® Universal blocking oligo-i5 (for six nucleotide indexes) (Integrated DNA Technologies). The recently released XGen® Universal Blocker – TS Mix (Integrated DNA Technologies) is also suitable for this application and it works under 6 and 8-base single or dual-indexing schemes. Targeted enrichment with large pools of biotinylated probes (i.e. >1,000 probes) worked satisfactorily with one round of capture. Hybridization capture experiments relying on a limited number of patient-specific probes demanded two rounds of targeted enrichment to achieve a desirable on-target rate^39^. Pools of libraries, Cot-1 Human DNA and blockers were dried out in a SpeedVac centrifuge and then resuspended, for at least 10 min, in 8.5 µl of 2x hybridization buffer (Integrated DNA Technologies), 2.7 µl of hybridization buffer enhancer (Integrated DNA Technologies) and 1.8 µl of ultra-pure water. Libraries were denatured at 95 °C during 10 min and mixed with 4 µl of personalized pools of xGen® Lockdown probes at 1 pmol/µl, 4 µl of the xGen® Pan-Cancer panel (Integrated DNA Technologies, see details about gene composition in https://www.idtdna.com/pages/products/nextgen/target-capture/xgen-lockdown-panels/xgen-pan-cancer-panel), 4 µl of our custom pool of lockdown probes targeting the exons of 72 lymphoma-related genes (see ref. 8 for details concerning the gene composition of our lymphoma-related gene pool) or 4 µl of our in-house generated DNA probes. Concerning personalized assays, we ensured, when possible, that targeted somatic mutations were centered within the probe. This strategy permits maximum coverage at the targeted site. We also ensured that targeted loci were of high complexity (i.e. there were no similar regions in the genome) and preferentially chose somatic mutations with high VAFs in the primary tumours to increase the odds to detect ctDNA when conducting personalized assays. For structural rearrangements, we designed personalized biotinylated probes spanning previously determined breakpoints. The hybridization reaction was incubated for a minimum of 4 hours and no more than 24 hours. Hybridized products were then capture using 75 µl of Streptavidin M-270 beads (Thermo Fisher Scientific) and subsequently washed following the xGen®rapid capture protocol version 2.1 (Integrated DNA Technologies). Washed beads were finally resuspended in 30 µl of ultra-pure water and captured hybridized fragments were amplified during 16 (double capture) or 12 (single capture) PCR cycles in a final volume of 70 µl containing 5 µl of Illumina P5 and P7 primers at 10 µM each and 35 µl of KAPA HiFi HotStart ready mix (Kapa Biosystems). We specifically extended the incubation time at 72 °C from 30 sec to 2 min per cycle (for personalized assays) and the final incubation time at 72 °C was extended to 4 min. Enriched libraries were finally measured in Qubit (large panels of probes) or subjected to a second round of targeted hybridization capture using this time half the amount of probes, Cot-1 DNA and blocking oligonucleotides^39^. The number of amplification cycles for the second post-capture PCR was reduced to 8 cycles. Dual-indexed libraries were sequenced in a MiSeq automated sequencer (Illumina) using PE 150 reads.

### Access Array Experiments on colorectal plasma samples

Patient specific simplex PCRs in the two colorectal patients were performed using the Fluidigm^®^ Access Array^™^ as per manufacturer’s protocols, with some modifications described below. All forward primers were tailed with the sequence CGCTCTTCCGATCTCTGNNNN, and all reverse primers were tailed with the sequence TGCTCTTCCGATCTGACNNNN, instead of tailing with common sequence tags (CS1 and CS2) to allow for easier use in downstream sequencing. Pre-amplification was performed on cfDNA using the manufacturer’s protocols with an input DNA volume of 5 μL at a concentration of ≥0.25ng/μL. A 21-plex (instead of 48-plex) primer mix (1 μM each primer) was used. Thermal cycling conditions were 95 °C (10 min), 15X[95 °C (15 sec), 60 °C(30 sec)], 4 °C(hold). ExoSAP-IT treated pre-amplification products were diluted by addition of 10 μL of dH_2_O.

Barcoding was performed using primers with sequences: forward, AATGATACGGCGACCACCGAGATCTACACTCTTTCCCTACACGACGCTCTTCCGATCTCT; reverse, CAAGCAGAAGACGGCATACGAGATXXXXXXGTGACTGGAGTTCAGACGTGTGCTCTTCCG (with the region denoted as XXXXXX reserved for sample indexes) instead of the Access Array Barcode Library in a 10 μL reaction. Products were pooled for sequencing using the Post-PCR Amplicon Purification and Quantitation protocol and sequenced on an Illumina^®^ MiSeq generating (typically) in excess of 50-fold coverage.

### Digital PCR

We employed patient-specific digital PCR assays to detect and quantify somatic mutations in the plasma of a subset of the patients investigated. For example, The *ALK-NEURL1* gene fusion detected in a neuroblastoma patient (NB-pt03) was tracked in plasma samples via a hydrolysis probe-based assay. We used primers 5′-GTAATCACAAAGTGGAGAGG-3′ (sitting on *ALK*) and 5′-CTGAATTCATTTATTAGTTCTCAGAG-3′ (sitting on *NEURL1*) and a hydrolysis probe spanning the fusion breakpoint (5′-/56FAM/TGGAGTTTTTTTTGCTAGCCAGACCTT/IABkFQ/-3′). An independent assay targeted the wild-type *ALK* sequence by using the same forward primer, an *ALK*-specific reverse primer (5′-CATGTAATCACAAAGTGGAG-3′) and a hydrolysis probe spanning the fusion breakpoint in the *ALK* wild-type sequence (5′-/56FAM/AGAAAGAGAATGCTAGCCAGACCTT/IABkFQ/-3′). Four digital PCR chips (two targeting the fusion breakpoint and another two targeting the wild-type version of the *ALK* gene) were amplified and analyzed using a QuantStudio^®^ 3D digital PCR system (Thermo Fisher Scientific) following the instructions recommended by the manufacturer.

Somatic mutations identified in the tumor biopsies of patients OSS-pt02 (osteosarcoma) and CPG-pt01 (craniopharyngioma) were traced in plasma samples through two single-probe hydrolysis-based assays. In essence, these assays leverage the high resolution provided by the QX200 Droplet Digital PCR system (Bio-Rad) to simultaneously quantify wildtype and mutant alleles with one single probe^24^. For OSS-pt02, we designed an assay targeting a *TBC1D8* somatic truncation (p.Q937*) with primers 5′-AGCACTCACTGAAAATGACC-3′ (FWD), 5′- CAGGGGTCTCGATGTTGA-3′ (REV) and a mutant-specific hydrolysis probe 5′-/56FAM/AGGATTCCTCAACGGCGACTGG/IABkFQ/-3′. For CPG-pt01, we designed an assay targeting a *CTNNB1* p.S26F somatic mutation with primers 5′-TTAGTCACTGGCAGCAAC-3′ (FWD), 5′-CTCAGAGAAGGAGCTGTG-3′ (REV) and a mutant-specific hydrolysis probe 5′-/56FAM/CCTGGACTTTGGAATCCATTCTGG/IABkFQ/-3′. Both assays were successfully validated on the QX200 Droplet Digital PCR system using tumor-derived DNA as positive controls and wild-type human DNA samples as negative controls before their use on plasma samples. PCR reactions were set up in a final volume of 22 µl following manufacturer’s recommendation and using the ddPCR Supermix for probes without dUTP (Bio-Rad). Hydrolysis probes were ordered as ZEN™ double-quenched probes from Integrated DNA Technologies.

We also tracked *TP53* p.R81X and *TP53* p.V41M somatic mutations in the plasma of the two breast cancer patients using standard hydrolysis probe-based assays (Neo 02-Fw:5′-AGGTCAAATAAGCAGCAGGAG-3′; Neo 02-Rv: 5′-GGAAATTTGCGTGTGGAGTATTT-3′; Neo 02 WT probe: 5′-/HEX/ACA + CTAT + GT + C + A + AAA + AGTIABkFQ/-3′; Neo 02 ALT probe: 5′-/6FAM/CA + CTAT + GT + C + G + AAA + AG/IABkFQ/-3′; Neo 027-Fw:5′-CTGCTCACCATCGCTATCTG-3′; Neo 027-Rv: 5′-ATGGCCATCTACAAGCAGTC-3′; Neo 027 WT probe: 5′-/HEX/CCT + C + A + G + AA + CCTCIABkFQ/-3′; Neo 027 ALT probe: 5′ 5′-/56FAM/CCT + C + A + C + AA + CC + TC/IABkFQ/-3′. Primers and probes were synthesized by Integrated DNA Technologies ( + stands for LNA modifications). For these specific assays, target preamplification assays were performed before ddPCR reactions.

### Recovery efficiency comparison between semi-degenerate barcoded adapters and standard sequencing adapters

One set of library replicates built from NB-pt01 cfDNA were built with 2.5 µl of a 20 µM stock (final concentration of each of the two oligoes that composed the adapter) of standard Y-shaped sequencing adapters for Illumina platforms (5′-ACACTCTTTCCCTACACGACGCTCTTCCGATC*T-3′; 5′-/5Phos/GATCGGAAGAGCACACGTCTGAACTCCAGTC-3′). The other three library replicates were built with 5 µl of a 45 µM solution of 12-nucleotide semi-degenerate adapters. Even though we used more barcoded than standard adapters it must be noted that a proportion of barcoded adapters are expected to not ligate, particularly those cases where a large number of annealing mispairings precede the 3′ overhanging thymine (see Supplemental File 1). Each library replicate was amplified using unique combinations of dual-indexed PCR primers and the pool of libraries was simultaneously enriched using five XGen® Lockdown probes during two rounds of capture^39^. Sequencing data was de-multiplexed in a MiSeq instrument (Illumina). On-target reads were filtered in Geneious ver 9.1.3. (Biomatters Ltd) by using an artificial reference built from the concatenation of the targeted loci plus 1 kb flanking sequence. Libraries were also subsampled to 152,000 reads, roughly the smallest number of on-target reads in any of the six libraries, in order to avoid any confounding effect of sample size on the number of recovered cfDNA molecules per library. The Picard MarkDuplicates tool (picard.sourceforge.net) was applied to on-target aligned reads using a custom installed plugin. Non-duplicated reads were extracted according to their mapping orientation and paired (unpaired reads were excluded). The resulting lists of paired reads were then merged using the BBMerge tool (ver 35.82) implemented in Geneious. Pairs of unmerged reads were individually assembled against the reference to generate a consensus sequence that included gaps for regions with no coverage in the reference sequence. The lists of merged reads and consensus sequences generated from unmerged reads were assembled against the concatenated reference. Reads that did not provide data at targeted sites (i.e. unmerged reads that showed gaps at those positions) were removed and the remaining sequences were used to count the number of unique molecules supporting either mutant or wildtype alleles.

### Analysis of barcode complexity across populations of ssDNA molecules

We estimated the diversity of our semi-degenerate barcode sequence in a subset of our cfDNA libraries (NB-pt01, OSS-pt01 and OVC-pt01 (V2)) using the fraction of on-target reads with overlapping ends (~75% of paired reads). Merged reads were mapped against the concatenated reference and sorted by read orientation. For each library, we extracted the reads that mapped against the reference in the forward (FWD) orientation and conducted a *de-novo* assembly in Geneious allowing for 1% mismatches, a maximum gap of 1 bp and 90 bp minimum overlap. We kept those consensus sequences supported by at least three independent reads. The consensus sequences displayed the most common base at any given position. The two molecular barcodes of each consensus sequence were aligned using the Geneious aligner, selecting the “create an alignment without actually aligning the sequences” option. Strand-specific tri-nucleotide tags were trimmed off. We discarded those consensus sequences (<2%) showing unexpected nucleotides or ambiguities at any of the 12 semi-degenerate barcode positions. The two 12-nucleotide barcodes per molecule were copied and concatenated in Microsoft Excel and the resulting lists of 24-nucleotide barcode sequences were re-imported into Geneious. We then used the “Find Duplicates” tool to extract unique sequences. The average number of nucleotide differences between UIDs was estimated from the matrix of pair-wise distances generated by the FastTree plugin installed in Geneious.

### Molecular barcoding analysis of dsDNA molecules

We analysed the three low-diversity NB-pt01 library replicates because we expected a low probability to sample more than one molecule sharing the same mapping coordinates. Consensus sequences built from at least 3 independent reads were mapped against the concatenated reference, annotated and sorted by their orientation in Geneious. Annotated sequences were imported in Microsoft Excel and renamed according to their mapping coordinates. This operation was performed independently for the two sets of consensus sequences exhibiting different orientations. Specifically for this analysis, we discarded those sequences sharing the same mapping coordinates and orientation (<12% of ssDNA consensus sequences). Consensus sequences from the putative two strands of a given molecule were individually analyzed by using the “assemble by name” option of the Geneious assembler. We used the high sensitivity settings, which allows for up to 50% mismatches, and set the maximum gap size to 0. This assembly is intended to generate a duplex consensus sequence from the two single stranded consensus sequences of each cfDNA molecule, as both may share mapping coordinates and thereby identical names, but it can also assemble the consensus sequences of two strands actually originating from different molecules. We found compelling support for this phenomenon in ~1% of the reconstructed duplexes (N = 17 cases) in NB-pt01. For these particular cases, the average number of mismatches between the two strands was 12.53 ± 3.12, mostly reflecting the average number of mismatches between two random 24-nucleotide UIDs (Fig. 2, Panel A). These molecules also exhibited a large number of base mispairings at the barcode positions immediately preceding the ligation sites, which may surely have precluded the successful ligation of an adapter with such characteristics (see Supplemental File 1). We therefore removed these cases from further analyses because they very likely represented situations in which we sampled two strands exhibiting different orientations but actually belonging to two different DNA molecules. Putatively “true” duplex consensus sequences were aligned using the Geneious aligner according to the option “create an alignment without actually aligning the sequences”. We exclusively selected those duplex consensus sequences that showed the expected nucleotides at each of the semi-degenerate barcode sites and then investigated the number and distribution of putative annealing mispairings (which were identified owing to the presence of ambiguities in the consensus sequence) as well as base composition ratios at each of the 12 barcode positions.

Recovery efficiencies of DNA duplexes were estimated as described below. Consensus sequences generated from at least three reads were mapped against the reference sequence. For each assembly, we extracted two independent lists containing ssDNA consensus sequences either in the forward (FWD) or reverse (REV) orientation. Tri-nucleotide fixed tags and the first 6 nucleotides of the semi-degenerate barcode were soft-trimmed. For each library, the smallest list of consensus sequences was mapped against the largest list of ssDNA consensus sequences using the low sensitivity settings of the Geneious “Map to Reference” tool but allowing for no gaps, a maximum of 1% mismatches and 90 bp minimum overlap (use existing trimmed regions option must be activated). The total number of unique molecules was calculated by summing the number of unassembled reads for the FWD and REV consensus sequence lists plus the number of putative duplex consensus sequences generated from this assembly. Duplex recovery rates were roughly estimated by dividing the number of putative DNA duplexes by the total number of molecules. For instance, if we start with 1,000 FWDs and 1,000 REVs consensus sequences and 600 duplexes are generated during the assembly, we then estimate ds recovery rates as 400 + 400 + 600 = 1,400 unique molecules; ds recovery rates = 600/1,400 = 0.4285.

### Error profiles and efficiency of our molecular barcoding method

We characterized the landscape of background errors that are corrected during single strand consensus sequencing by searching for discrepancies between consensus sequences and individual reads in 10,000 assemblies generated from both OSS-pt01 and OVC-pt01 (V2) sequence data. We also mapped single-strand consensus sequences generated from at least three merged reads and searched for non-reference alleles in Geneious. Single stranded consensus sequences kept the most common bases at each position. Those positions showing ambiguities in the consensus sequence (occurring when two variants are found at equal proportions) were not considered during the total count of non-reference allele calls. Duplex consensus sequences were generated as described above and showed ambiguous bases at those positions where disagreements between the consensus sequences of individual strands existed. Such ambiguities were not considered as non-reference allele calls either. We soft-trimmed the first two nucleotides after the ligation sites following evidence from a previous study that shows a high incidence of errors across these regions associated with the action of end-repair enzymes^23^. We still observed, however, a minute number of cases where the ends of the molecules might have been affected by some sort of artifact during library prep. These artifacts involved a relatively large number of mismatches when compared to the reference, and as such, they were easily identified and discarded when calculating background noise statistics.

### Analysis and error-correction of cfDNA sequencing data through user-friendly bioinformatics workflows

Our Geneious-based error correction method for barcoded cfDNA libraries subjected to targeted enrichment using personalized probes relies on three separate analytical workflows. The two first workflows (cfDNA-GenWkf1 and cfDNA-GenWkf2) filter on-target reads and generate consensus sequences from paired reads with and without overlapping ends, respectively. All loci from a given personalized experiment are concatenated in Geneious to produce the personalized reference (see Supplemental File 2 for more details). Targeted sites are annotated in the reference as “Somatic mutations”. For those loci with low genomic complexity we also included regions with more than >90% similarity in the human reference genome. The third workflow (cfDNA-GenWkf3) assembles the consensus sequences against the reference sequence and searches for variants specifically across those sites labeled as “Somatic mutations”. Duplex support can be visually inspected in Geneious by sorting the contig by base at each target position. The workflows that analyse sequencing data from enrichment experiments with large gene panels rely on essentially the same algorithm but incorporate a few important modifications. First, there is no minimum number of reads to generate any consensus sequences and non-assembled reads are retained except those filtered out due to low quality. Second, variant calls are not restricted to specific sites and we searched for non-reference alleles across coding regions and splice donor/acceptor sites. The workflows mentioned above can be downloaded directly from the supplemental material. Workflows intended to work on large captured regions have the suffix -l added to the names of the three main workflows described above. We noted that this intuitive and straightforward bioinformatics workflow performs well on relatively simple scenarios like the ones presented here but scalable and extensible open-source computational workflows may be better suited for the analysis of complex and larger data sets^21–23, 40^. To address this, we have also developed software (ProDuSe)^8^ and provide the source code at the following location: https://github.com/morinlab/ProDuSe.

## Electronic supplementary material

Supplemental Figures and Tables

Supplemental Information

Supplemental Information

Supplemental Information
