# Supplementary material for: Targeted error-suppressed quantification of circulating tumor DNA using semi-degenerate barcoded adapters and biotinylated baits
Source: Sci Rep. 2017 Sep 5;7:10574. doi: 10.1038/s41598-017-10269-2 (PMC5585219; doi:10.1038/s41598-017-10269-2)
Supplement: Supplementary file 1 — Supplemental Figures and Tables [file 41598_2017_10269_MOESM1_ESM.pdf]

SUPPLEMENTARY INFORMATION

Targeted error-suppressed quantification of circulating tumor DNA using semi-degenerate barcoded adapters and biotinylated baits

AUTHORS

Miguel Alcaide[1], Stephen Yu[1], Jordan Davidson[1], Marco Albuquerque[1], Kevin Bushell[1], Daniel Fornika[1], Sarah Arthur[1], Bruno Grande[1], Suzan McNamara[2], Mathilde Couetoux du Tertre[2], Gerald Batist[2], David G. Huntsman[3], Luca Cavallone[4], Adriana Aguilar[4], Mark Basik[4], Nathalie A. Johnson[4], Rebecca J. Deyell[5], Rod Rassekh[5], and Ryan D. Morin[1].

FIGURE S1. In-house generation of custom DNA biotinylated baits for ultra-targeted enrichment of cfDNA libraries. Small genomic regions (~120 bp) known to harbor somatic mutations in the primary tumours are amplified with locus-specific primers carrying M13 tails during 15 cycles of PCR (1). The second round of amplification involves the use of universal 5’-biotinylated and 5’-phosporylated M13 primers during 35 cycles (2). PCR amplicons are then incubated with lambda exonuclease, which specifically degrades the amplicon strands that are phosphorylated at the 5’ end, leaving single-stranded 5’-biotinylated baits ready to be used after DNA purification with magnetic beads (3).

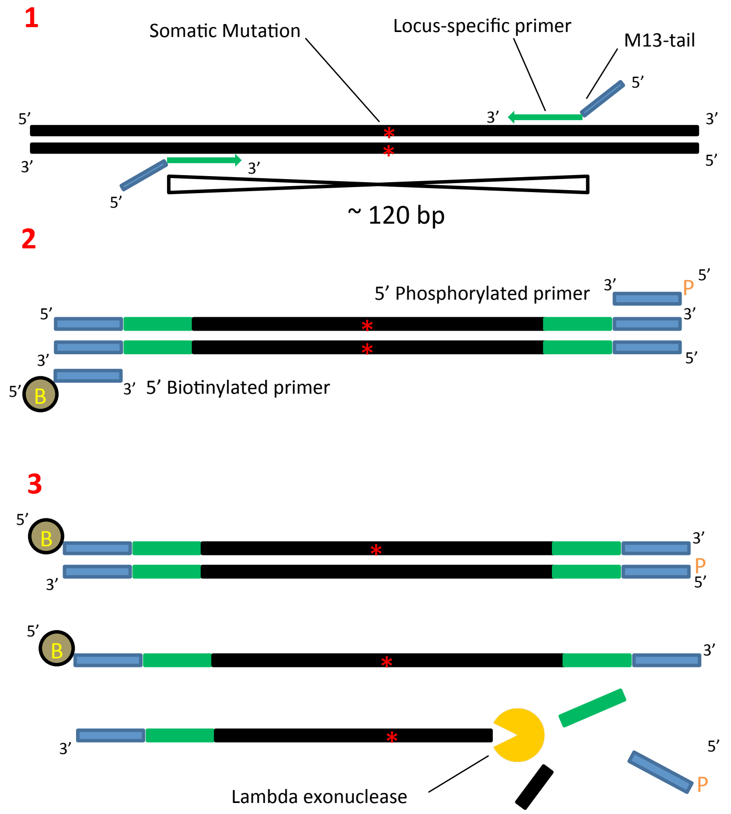

FIGURE S2. Distribution of sequencing coverage across targeted loci in two independent hybridization capture experiments. The first library (OVC-pt01 (V2)) was enriched with six commercially synthesized XGen© Lockdown© biotinylated baits. The second library (OSS-pt01) was enriched with four DNA biotinylated baits produced in-house. Both libraries were enriched through two successive rounds of hybridization capture.

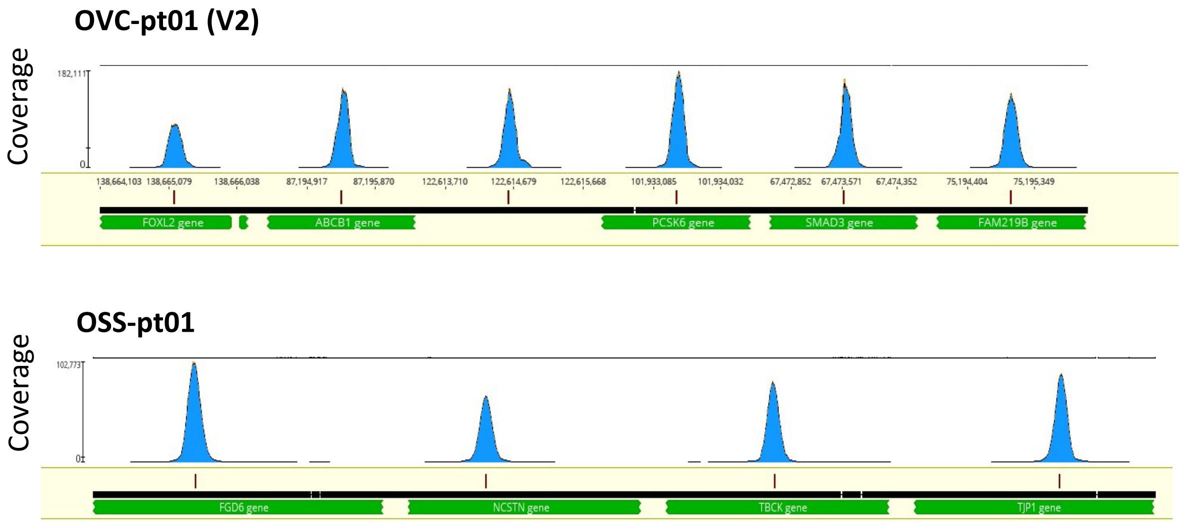

FIGURE S3. Extreme mapping coordinate collision in the cfDNA library built from OVC-pt01 (V2) plasma. This figure shows as many as 63 molecules carrying distinct UIDs (12-nucleotide semi-degenerate barcodes are colored) but sharing the same mapping coordinates. Only one of the two strands (plus) is represented in this figure (i.e. all molecules share the same strand-specific adapter tag). This figure also permits the appreciation of three C>A artifacts that were resilience to ssDNA consensus sequencing. One common SNP (C/T) can be noticed within the targeted region.

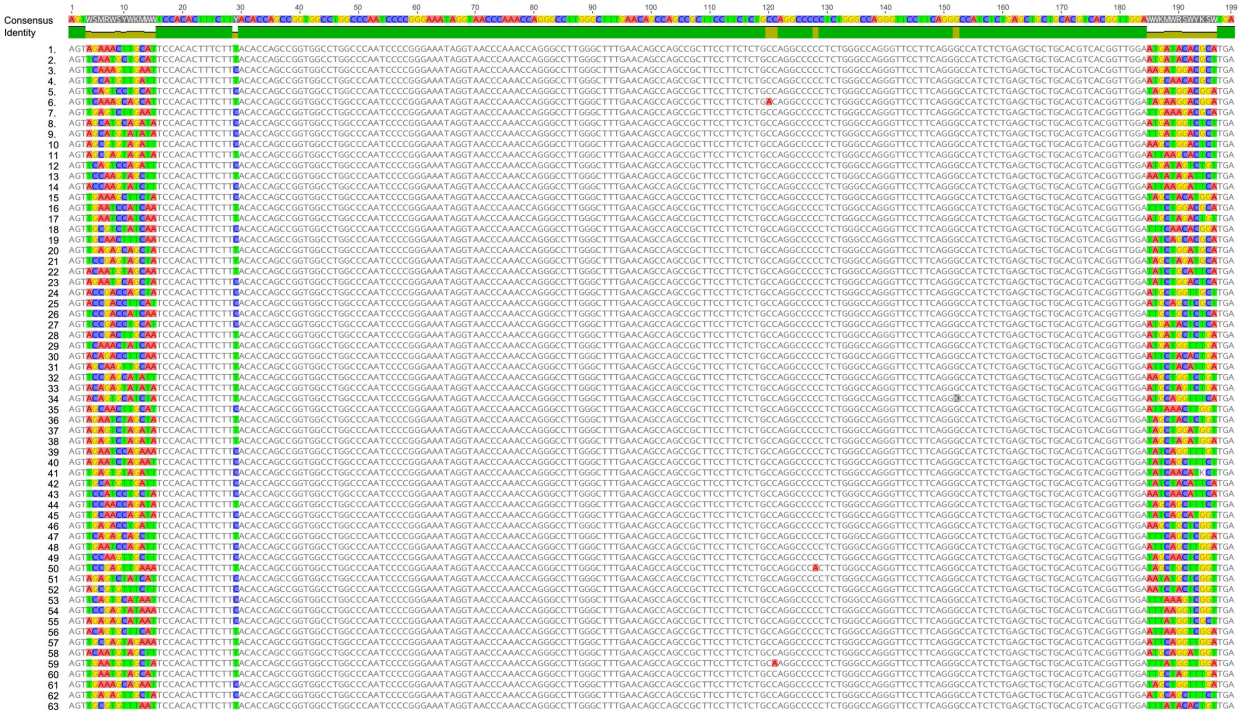

FIGURE S4. Another example of mapping coordinates collision showing as many as 12 unique molecules represented by the consensus sequence of the two parental strands in the library built from OSS-pt01 plasma (Panel A). An additional molecule with a mapping coordinate differing in just one nucleotide is also included for comparative purposes. Note that all but one of these molecules (with one annealing artifact) showed no annealing mismatches along the barcode region preceding the ligation site (N=6+6=12 nucleotides). All these molecules support the wild-type allele of one of the targeted loci in this patient. Panel B shows a mapping coordinate collision between two cfDNA molecules with unique barcodes but supporting either the wildtype (A) or the mutant allele (G). Note again that the two parental strands were sampled in both cases and that no annealing artifacts were observed along the 12-nucleotide barcode preceding the ligation site.

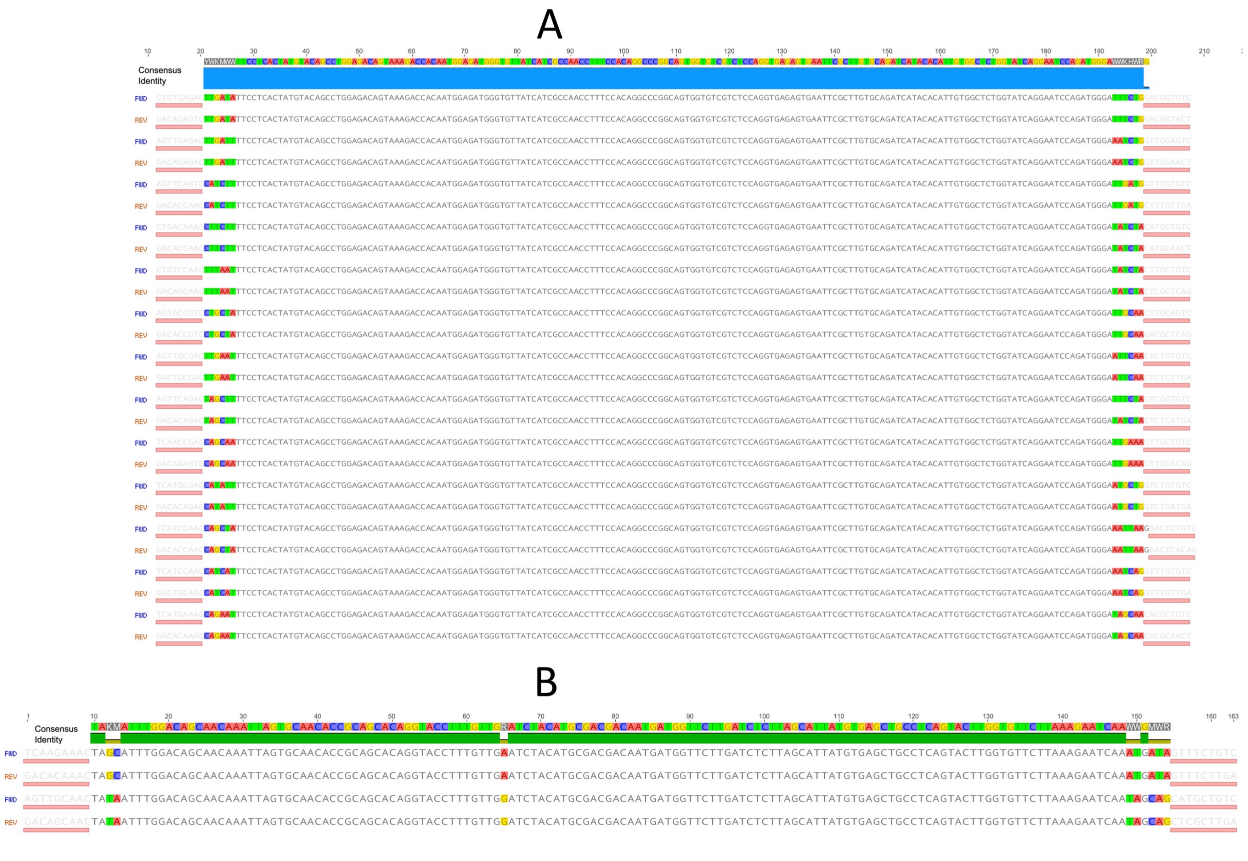

FIGURE S5. Error-correction of strand-specific artifacts. Some variants are exclusively present in many, if not all, copies of the PCR families derived from one single strand and not observed in any of the copies of the complementary strand. Strong imbalances in these artifacts resistant to single strand consensus sequencing (e.g. C>A versus reciprocal G>T) indicate that oxidative damage mainly occurs during the hybrid capture step, as it preferentially targets the minus strand of our libraries (i.e. the strand that hybridizes against the biotinylated plus strands used during our enrichment experiments). Nucleotide discrepancies are colored in the assemblies here presented, with the reference sequences shown on top. The three first and last nucleotide positions correspond to the strand-specific tags and are therefore different in the two sets of reads showing different orientation (i.e. either FWD or REV). Disagreements associated with barcode annealing mispairings, punctual artifacts caused by either sequencing or PCR errors and even SNPs strongly supported by the two parental strands (examples 2 and 3) can be also noticed.

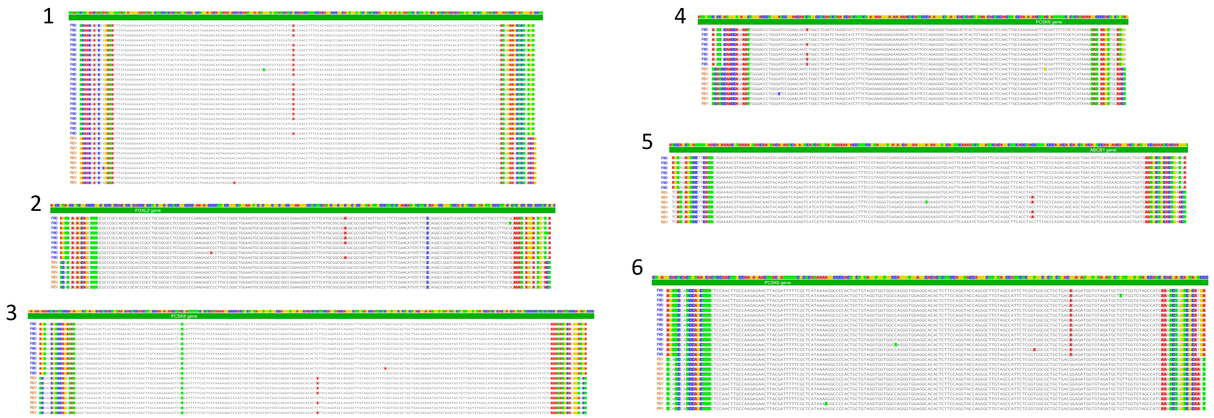

FIGURE S6. Tracking strand-specific DNA damage in cfDNA-derived libraries. Oxidative DNA damage leads to 8-oxoguanine and cytosine deamination. For example, oxidized 8-oxoguanine nucleotides (orange bases) show high affinity for adenine (red bases) but can still pair with cytosine (blue bases) during PCR replication. DNA damage occurring before library preparation is expected to generate a balanced ratio of G>T to C>A artifacts, as damage may occur randomly in any of the two strands. Ours and previous studies[21] have documented strong imbalances in these ratios during targeted hybridization capture experiments. This phenomenon can be explained by oxidative damage of just one of the library-derived strands, i.e. either the plus or the minus strand (depending on the orientation of the biotinylated bait employed during the enrichment step).

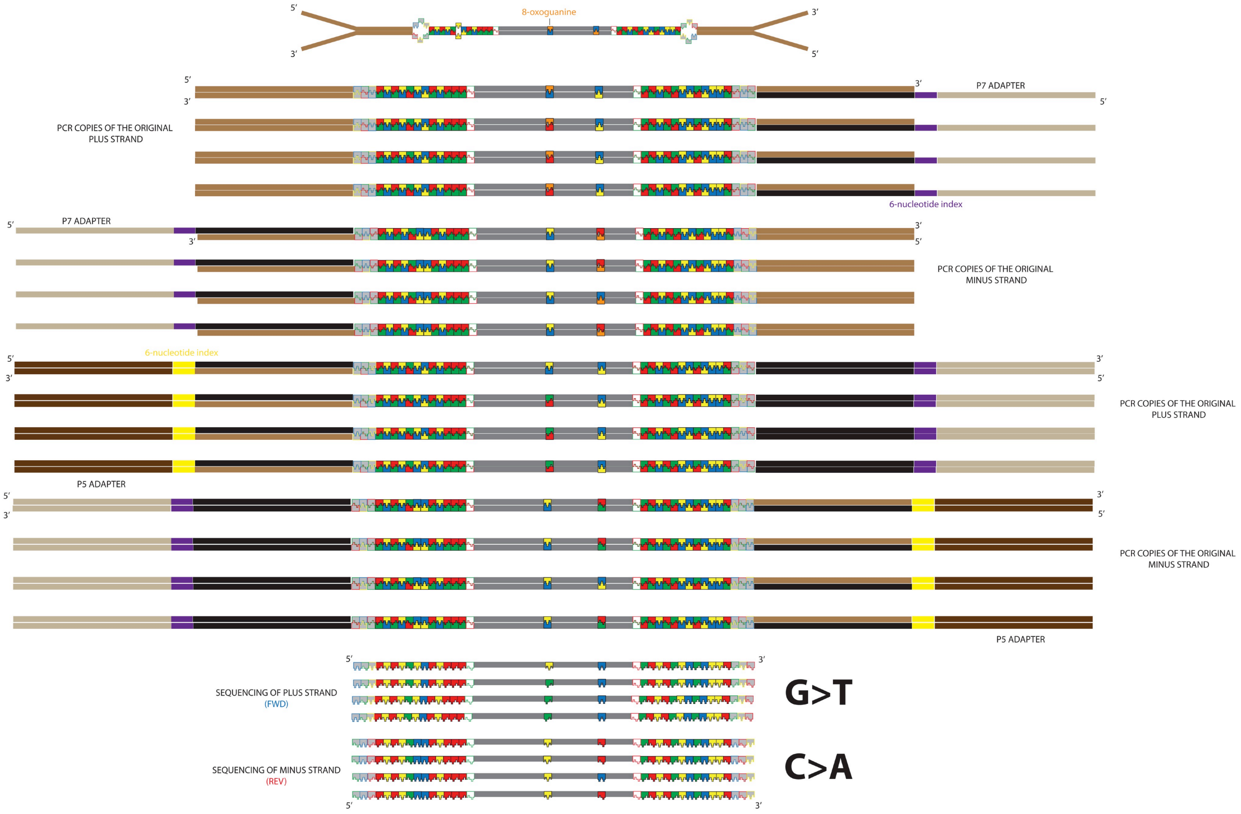

FIGURE S7. A. Correlation between the variant allele frequencies (VAF) for *APC*, *TP53* and *KRAS* somatic mutations, estimated through amplicon sequencing and targeted hybridization capture approaches, in the plasma of two colorectal cancer patients. **B.** Correlation between the VAF of *TP53* somatic mutations, as inferred by targeted hybridization capture and ddPCR, in the same set of six plasma samples from two breast cancer patients. Two samples with VAFs of 0.0012 and 0.0003 were positive by ddPCR and negative for targeted hybrid capture. This result can be explained by the limited number of cfDNA molecules analyzed in the two negative cfDNA-derived libraries.

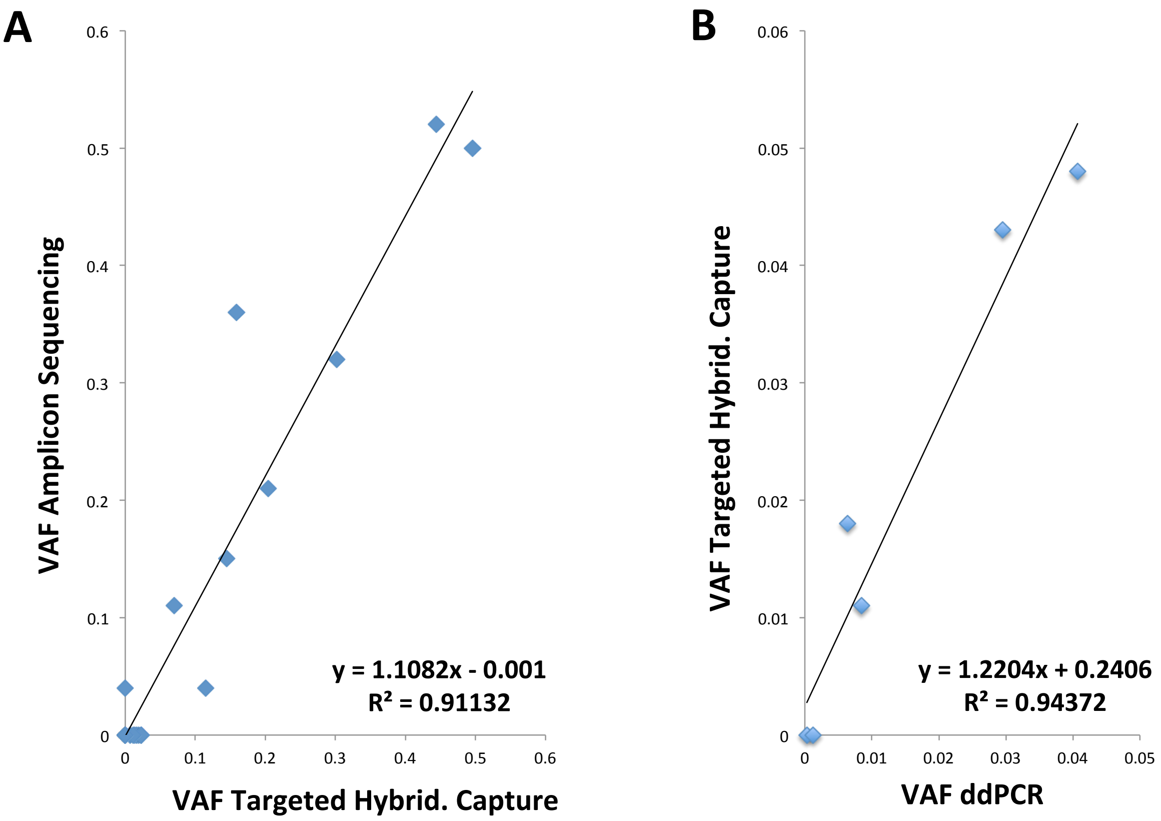

TABLE S1. Genomic coordinates of the somatic mutations targeted in the cohort of cancer patients investigated in this study. In addition to personalized biotinylated baits, we also used commercially available gene panels (e.g. XGen® Pan-Cancer Gene Panel, Integrated DNA Technologies) and/or our custom lymphoma-related gene panel built from pools of individual XGen® lockdown probes (Integrated DNA Technologies) in certain patients. Those genomic sites supporting the presence of ctDNA are highlighted in red.

OSS-pt01 (OSTEOSARCOMA) (POG)
Chr12:95486541 c>g FGD6
Chr1:160326480 c>t NCSTN
Chr4:107133967 c>g TBCK
Chr15:30011989 g>a TJP1

NB-pt03 (NEUROBLASTOMA) (POG)
Chr9: 140850167 g>a CACNA1B
Chr16: 83998770 g>t OSGIN1
Chr5: 176794018 c>t RGS14
Chr11: 47431848 g>c SLC39A13
NEURL-ALK FUSION (10:105285095-2:29643745)

SAR-pt01 (SARCOMA) (POG)
EWSR1-ATF1 FUSION (22:29287535-12:50813322)

HGL-pt01 (HODGKIN LYMPHOMA) (POG)
XGen Pan-Cancer Panel (IDT) + CUSTOM LYMPHOMA PANEL
Chr17:70119084 g>a SOX9
Chr17:7577518 t>a TP53
Chr12:49426774 t>a KMT2D
Chr6: 393205 g>a IRF4
Chr4: 55594095 t>a KIT
Chr4: 103488256 g>a NFKB1
Chr3: 176767928 t>a TBL1XR1
Chr12: 78513679 c>g NAV3
Chr8: 55371693 a>t SOX17

OVC-pt01 (OVARIAN GRANULOSA) (V1) (POG)
Chr3:138665163 g>c FOXL2
Chr7:87195412 c>a ABCB1
Chr15:101933416 c>t PCSK6
Chr15:67473606 c>t SMAD3
Chr15:75195028 t>c FAM219B
Chr12:122614621 c>t MLXIP

DLBCL-pt01 (DLBCL) (POG)
Chr3:133894782 c>a RYK
Chr7:157151332 g>a DNAJB6
Chr4:72316187 g>a SLC4A4
Chr1: 60373527 c>t CYP2J2

ALL-pt01 (ACUTE LYMPHOBLASTIC LEUKEMIA) (POG)
XGen Pan-Cancer Panel (IDT)
Chr4:55561752 g>c KIT
Chr10: 112356279 t>g SMC3
Chr12: 25398284 c>g KRAS

CCR-pt029 (COLORECTAL CANCER)
XGen Pan-Cancer Panel (IDT)
Chr5:112176022 ->ta APC
Chr5:112128143 c>t APC
ChrX:76949318 c>t ATRX
Chr17:63554663 c>t AXIN2
Chr1: 201981307 ->a ELF3
Chr4:153247288 c>t FBXW7
Chr12:25378561 g>a KRAS

Neo-02 (BREAST CANCER)
TP53 GENE PANEL
Chr17:7578212 g>a TP53

Neo-027 (BREAST CANCER)
TP53 GENE PANEL
Chr17:7578413 c>g TP53

OVC-pt01 (OVARIAN GRANULOSA) (V2) (POG)
Chr3:138665163 g>c FOXL2
Chr7:87195412 c>a ABCB1
Chr15:101933416 c>t PCSK6
Chr15:67473606 c>t SMAD3
Chr15:75195028 t>c FAM219B
Chr12:122614621 c>t MLXIP

OVC-pt02 (OVARIAN CANCER) (POG)
Chr4:86952560 g>a MAPK10
Chr7:76111953 g>t DTX2
Chr8:119964030 c>t TNFRSF11B
Chr11: 10615663 g>a MRVI1
Chr18:43310288 g>a SLC14A1

NB-pt04 (NEUROBLASTOMA) (POG)
Chr4:74351771 a>t AFM
Chr5:159520751 g>t PWWP2A
Chr12: 4874639 c>t GALNT8
Chr13:73355142 c>a DIS3
Chr10:5810192 del TTGGTTCTGTGGAA GDI2

ESR-pt01 (EWING SARCOMA) (POG)
XGen Pan-Cancer Panel (IDT)
Chr17:7578492 c>t TP53

IFB-pt01 (INFANTILE FIBROSARCOMA) (POG)
Chr3:1394006 a>g CNTN6

MGC-pt01 (MALIGNANT GRANULLAR CELL TUMOUR) (POG)
Chr 11:102819865 g>a MMP13

ASL-pt01 (ANGIOSARCOMA OF LIVER) (POG)
Chr7:81978944 c>a CACNA2D1
Chr2: 25398255 g>t KRAS
Chr6: 161771131 c>g PARK2
Chr19:4839394 g>a PLIN3

PIB-pt01(CEREBELLUM TUMOR) (POG)
Chr17:7578535 t>c TP53

NB-pt02 (NEUROBLASTOMA) (POG)
Chr6: 44140067 ->GGCTGCC CAPN11

CCR-pt049(COLORECTAL CANCER)
XGen Pan-Cancer Panel (IDT)
Chr17:7577018-7577026 cctcgctta>- TP53
Chr17:7577043 g>- TP53
Chr12:25398285 c>a KRAS

NMC-pt01 (NUT MIDLINE CARCINOMA) (POG)
NUTM1-BRD4 FUSION (15:34637730-15359588)

DLBCL-pt015 (DIFUSSE LARGE B-LYMPHOMA)
CUSTOM LYMPHOMA PANEL
Chr13:41240322 t>c
Chr13:41240349 t>c

TABLE S2. Results of the targeted hybridization capture experiments, using a panel of lymphoma-related genes, conducted on a set of libraries built from serial dilutions of a DB cell line DNA carrying an *EZH2* Y641N mutation. VAF stands for inferred allele frequencies of mutant versus wild-type DNA. We spiked in two different amounts of non-fragmented wild-type genomic DNA (HMW-gDNA), accounting for 10% or 75% of total DNA mass, across two parallel sets of serial dilutions of this cell line DNA (Top Table or "A"). Equivalent cell line dilutions from the two treatments were eventually pooled for the determination of VAFs and absolute mutant molecule quantification given that we did not observe significant differences between the two treatments (Bottom Table or "B"). This is due to the fact that non-fragmented DNA is not amenable for library construction and therefore does not get sequenced. Digital PCR[34] suggest a 3/2 ratio for mutant versus wild-type alleles, an observation that is consistent with our serial dilution data and a documented copy number alteration affecting chromosome 7 in pure samples of this cell line DNA.

A

DB CELL LINE DILUTION	VAF Plus 10% HMW-gDNA	VAF Plus 75% HMW-gDNA	Total Molecule Count Plus 10% HMW-gDNA	Total Molecule Count Plus 75% HMW-gDNA
1/2 DILUTION	0.329	0.395	200	214
1/5 DILUTION	0.152	0.155	273	193
1/50 DILUTION	0.023	0.013	256	150
1/500 DILUTION	0	0.005	254	193

B

DB CELL LINE DILUTION	VAF Expected	VAF Observed	Mutant Molecule Count	Total Molecule Count
1/2 DILUTION	0.3	0.359	149	414
1/5 DILUTION	0.12	0.152	71	466
1/50 DILUTION	0.012	0.019	8	406
1/500 DILUTION	0.0012	0.0022	1	447

TABLE S3. DNA sequences of 10 oligonucleotides (4 “minus/r” and 6 “plus/f”) suitable for the generation of up to 19 non-complementary (across the tri-nucleotide fixed tag region) and unique adapter combinations amenable for duplex sequencing. Preferred fixed tag combinations in terms of non-complementarity and nucleotide diversity are highlighted in green color. Oligonucleotide combinations that generate perfectly self-complementary fixed tags are avoided and indicated by X. DNA sequences are given in the 5’ to 3’ direction.

Ad-12nt-TagPlus1r (Fixed tag: GTC)

/5Phos/WWKMWRSWYKSWGTCAGATCGGAAGAGCACACGTCTGAACTCCAGTC

Ad-12nt-TagPlus2r (Fixed tag: TGA)

/5Phos/WWKMWRSWYKSWTGAAGATCGGAAGAGCACACGTCTGAACTCCAGTC

Ad-12nt-TagPlus3r (Fixed tag: ACT)

/5Phos/WWKMWRSWYKSWACTAGATCGGAAGAGCACACGTCTGAACTCCAGTC

Ad-12nt-TagPlus4r (Fixed tag: CAG)

/5Phos/WWKMWRSWYKSWCAGAGATCGGAAGAGCACACGTCTGAACTCCAGTC

Ad-12nt-TagPlus1f (Fixed tag: ATG)

ACACTCTTTCCCTACACGACGCTCTTCCGATCTATGWSMRWSYWKMWW*T

Ad-12nt-TagPlus2f (Fixed tag: CCT

ACACTCTTTCCCTACACGACGCTCTTCCGATCTCCTWSMRWSYWKMWW*T

Ad-12nt-TagPlus3f (Fixed tag: AGT)

ACACTCTTTCCCTACACGACGCTCTTCCGATCTAGTWSMRWSYWKMWW*T

Ad-12nt-TagPlus4f (Fixed tag: CTG)

ACACTCTTTCCCTACACGACGCTCTTCCGATCTCTGWSMRWSYWKMWW*T

Ad-12nt-TagPlus5f (Fixed tag: TCA)

ACACTCTTTCCCTACACGACGCTCTTCCGATCTTCAWSMRWSYWKMWW*T

Ad-12nt-TagPlus6f (Fixed tag: GAC)

ACACTCTTTCCCTACACGACGCTCTTCCGATCTGACWSMRWSYWKMWW*T

		5f	3f	4f	1f	2f	6f	
		**TCA**	**AGT**	**CTG**	**ATG**	**CCT**	**GAC**	**PLUS**
1r	**GTC**	TCA/GTC	AGT/GTC	CTG/GTC	ATG/GTC	CCT/GTC	**X**	
2r	**TGA**	**X**	AGT/TGA	CTG/TGA	ATG/TGA	CCT/TGA	GAC/TGA	
3r	**ACT**	TCA/ACT	**X**	CTG/ACT	ATG/ACT	CCT/ACT	GAC/ACT	
4r	**CAG**	TCA/CAG	AGT/CAG	**X**	**X**	CCT/CAG	GAC/CAG	
	**MINUS**							
