# Supplementary material for: Targeted error-suppressed quantification of circulating tumor DNA using semi-degenerate barcoded adapters and biotinylated baits
Source: Sci Rep. 2017 Sep 5;7:10574. doi: 10.1038/s41598-017-10269-2 (PMC5585219; doi:10.1038/s41598-017-10269-2)
Supplement: Supplementary file 3 — Supplemental Information [file 41598_2017_10269_MOESM3_ESM.pdf]

GENEIOUS DETAILED WORKFLOW FOR THE ANALYSIS OF CELL-FREE DNA LIBRARIES SUBJECTED TO TARGETED HYBRIDIZATION CAPTURE USING PERSONALIZED BIOTINYLATED BAITS AND NGS

1. Import Fast Q files into Geneious.

1.1. Drag FastQ files into Geneious or
1.2. Go to File > Import > From File
1.3. Set the two sequence lists (_R1 and _R2) as paired reads. Go to Sequence > Set Paired Reads. Set expected distance/insert size to 300 bp. Delete the two original FastQ files
1.4. Set up Reference
1.5. Download Human Reference Genome and import it into Geneious (e.g. http://support.illumina.com/sequencing/sequencing_software/igenome.html)
1.6. Label targeted positions (e.g. those genomic positions mutated in the primary tumors) as "**Somatic Mutations**". Select a base or group of bases in the reference and then click on the "**Add Annotation**" button.

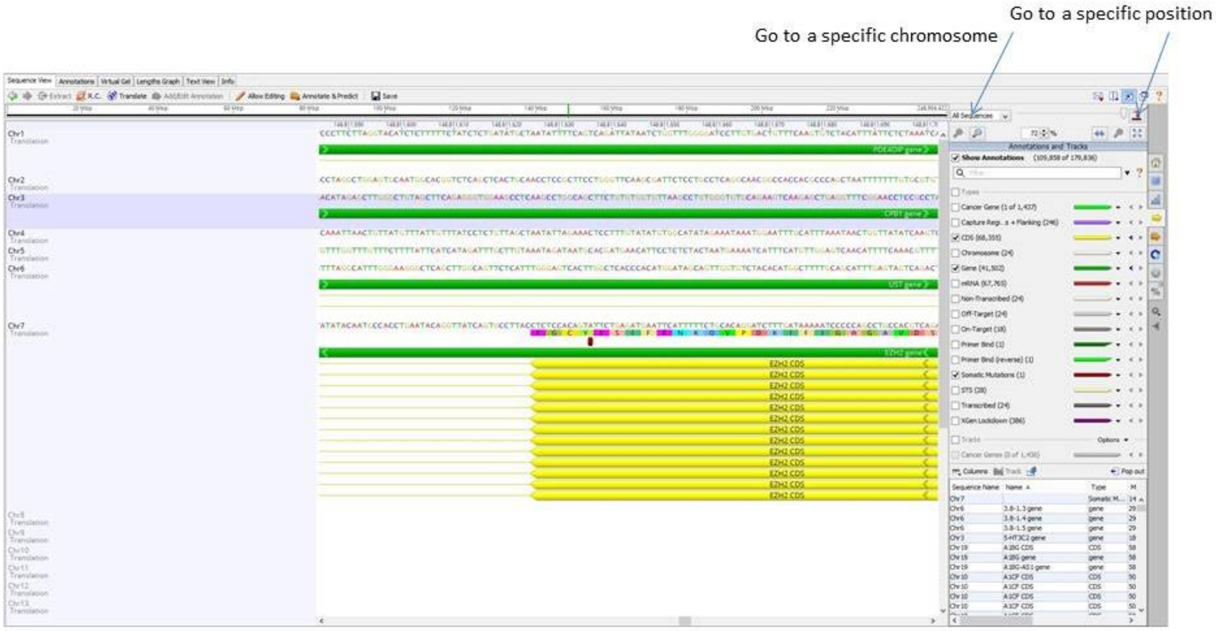

1.7. Go to Tools > Extract Annotations and use the following settings:

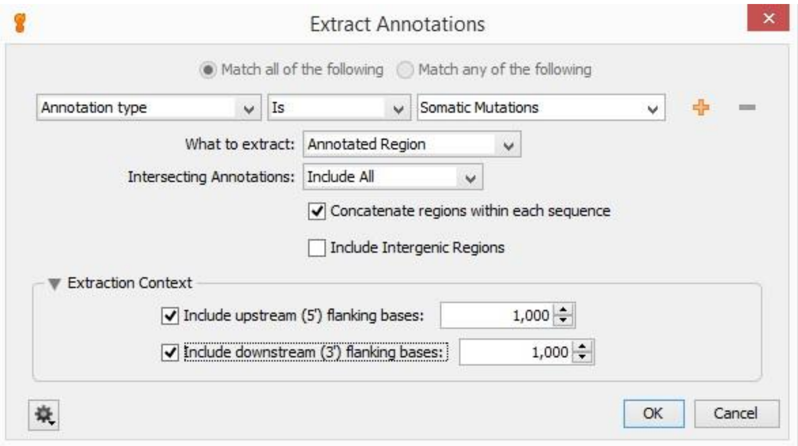

1.7. Select all extractions (Ctrl+A) and then go to Tools>**Concatenate Sequences** or Alignments. Add 500 bp spacers between loci. Do not circularize sequences.

1.8. If selected loci display high sequence complexity (there are no other regions of the genome with >90% sequence similarity) the reference is finished. Otherwise, similar loci must be included in the reference to avoid mapping artifacts. Running local **MegaBLAST** in Geneious or other platforms (i.e. UCSC Blat tool, https://genome.ucsc.edu/cgi-bin/hgBlat?command=start) can help ascertaining weather loci are composed of unique sequences or if similar loci (i.e. paralogs, pseudogenes etc.) exist. If so, extract those loci and concatenate them into the reference. Use the % Pairwise Identity and the Query Coverage Scores, which should be <90%.

2. Select reference and list of paired reads and go to **Workflows > cfDNA-GenWkf1**

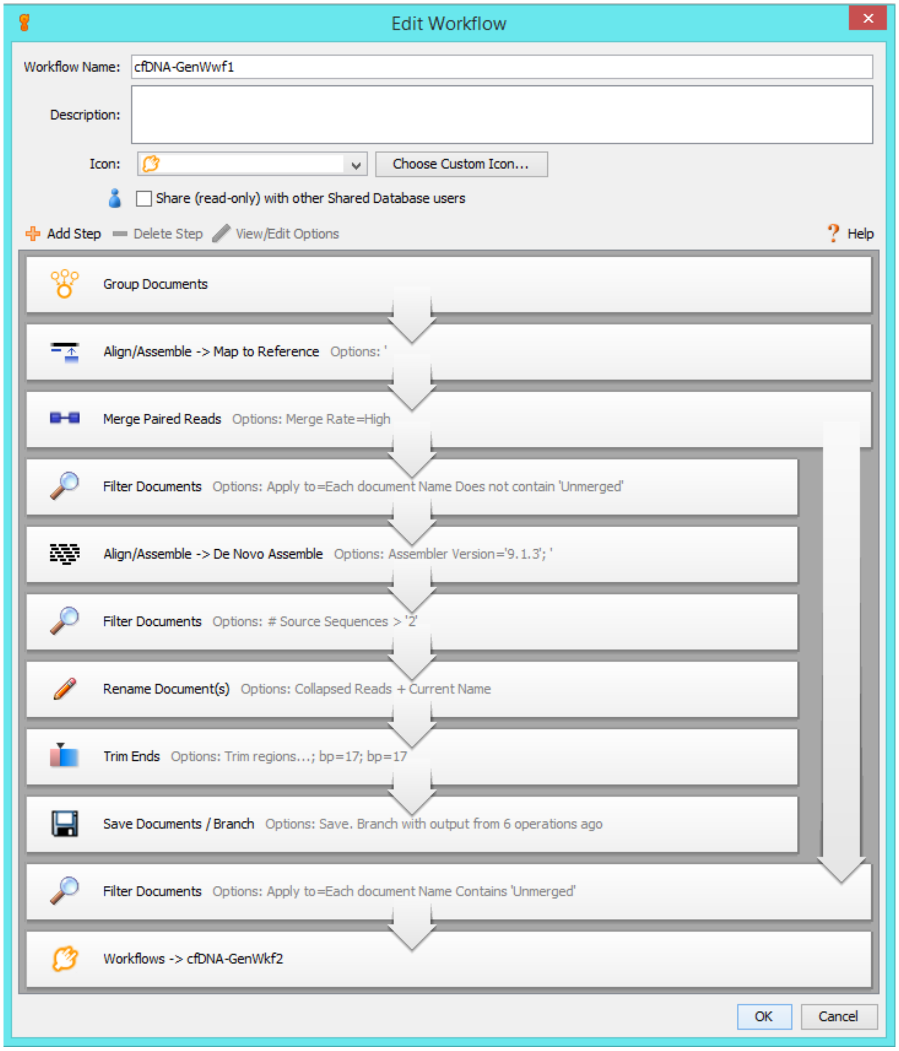

*Customizable options for the *Align/Assemble – Map to Reference* step of the workflow

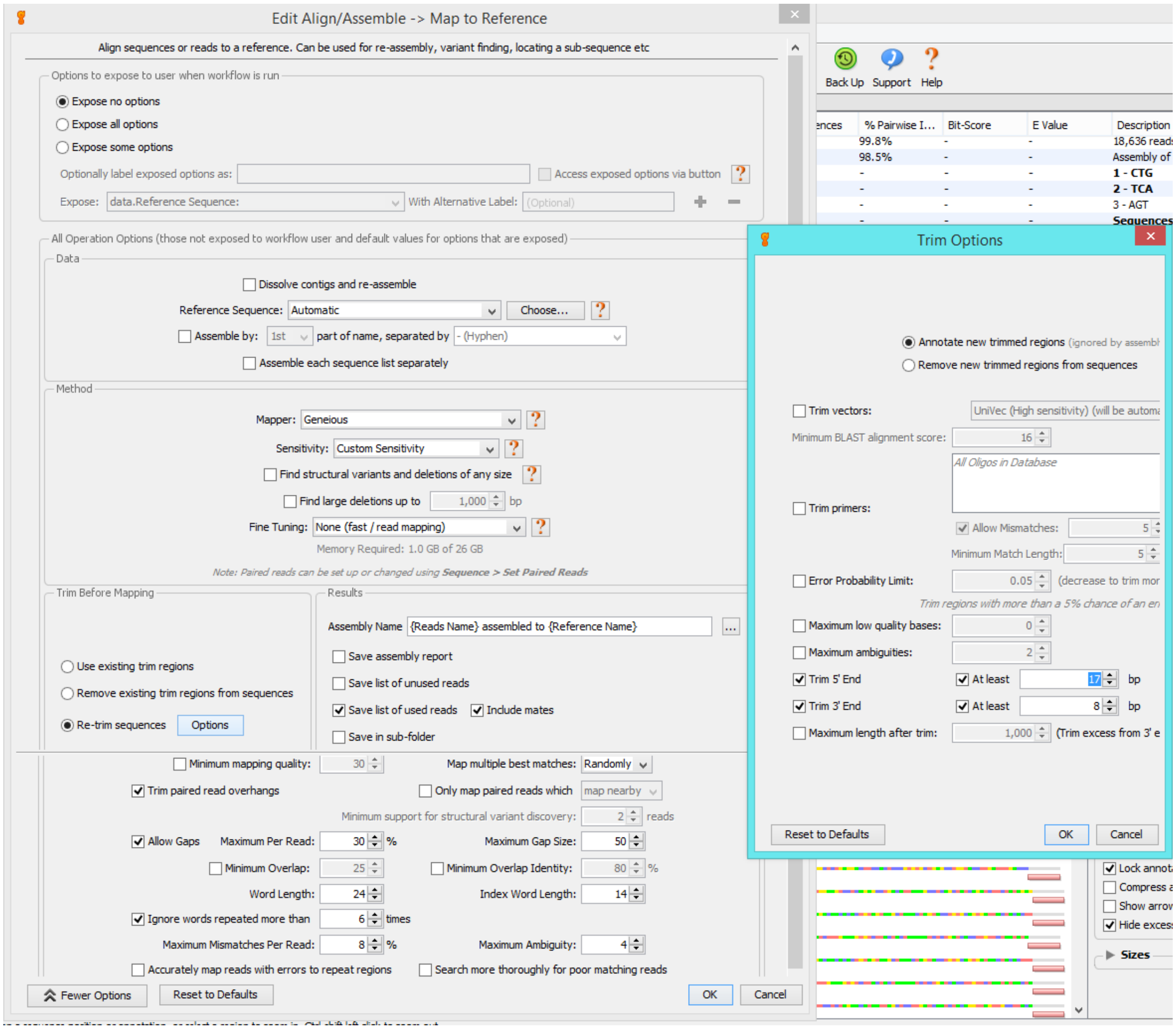

*Customizable options for the *De Novo Assemble* step of the workflow

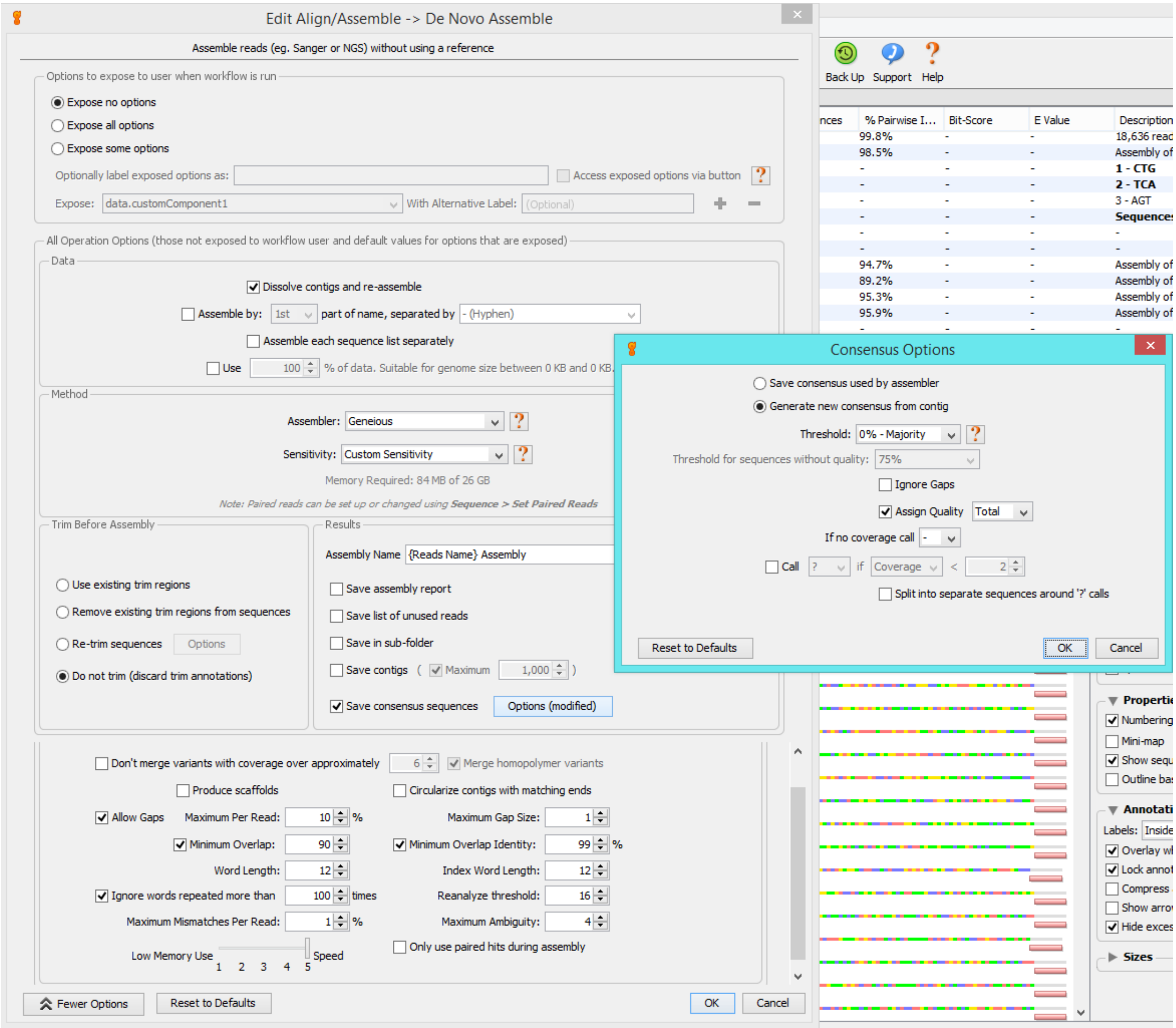

*Word Length and Index Word Length are the parameters that may stronger influence the speed of this operation.

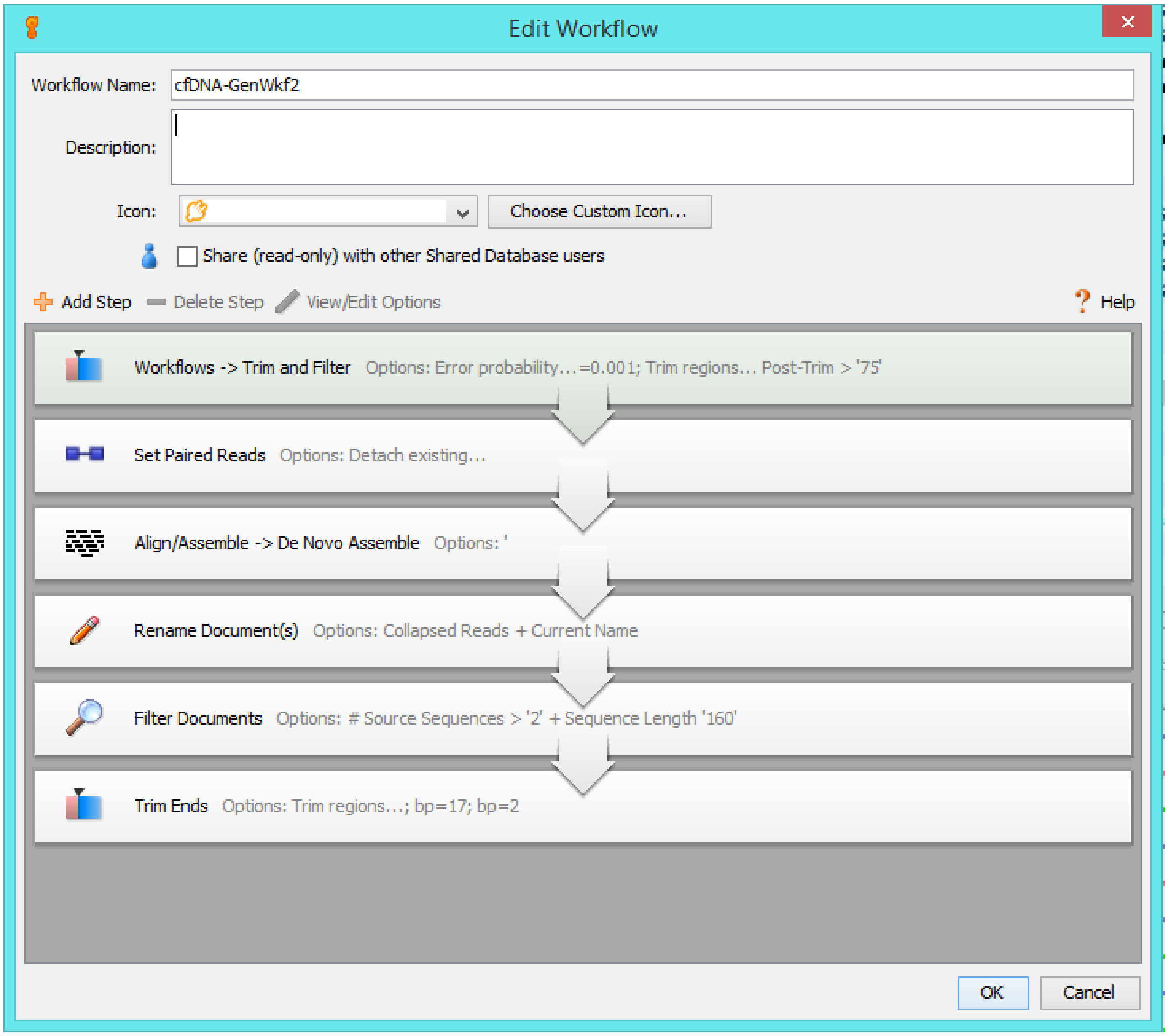
Edit Workflow
Workflow Name: cfDNA-GenWkf2
Description:
Icon:
Choose Custom Icon...
Share (read-only) with other Shared Database users
Add Step
Delete Step
View/Edit Options
Help
Workflows -> Trim and Filter Options: Error probability...=0.001; Trim regions... Post-Trim > '75'
Set Paired Reads Options: Detach existing...
Align/Assemble -> De Novo Assemble Options: '
Rename Document(s) Options: Collapsed Reads + Current Name
Filter Documents Options: # Source Sequences > '2' + Sequence Length '160'
Trim Ends Options: Trim regions...; bp=17; bp=2
OK
Cancel

*Customizable options for the *De Novo Assemble* step of the workflow

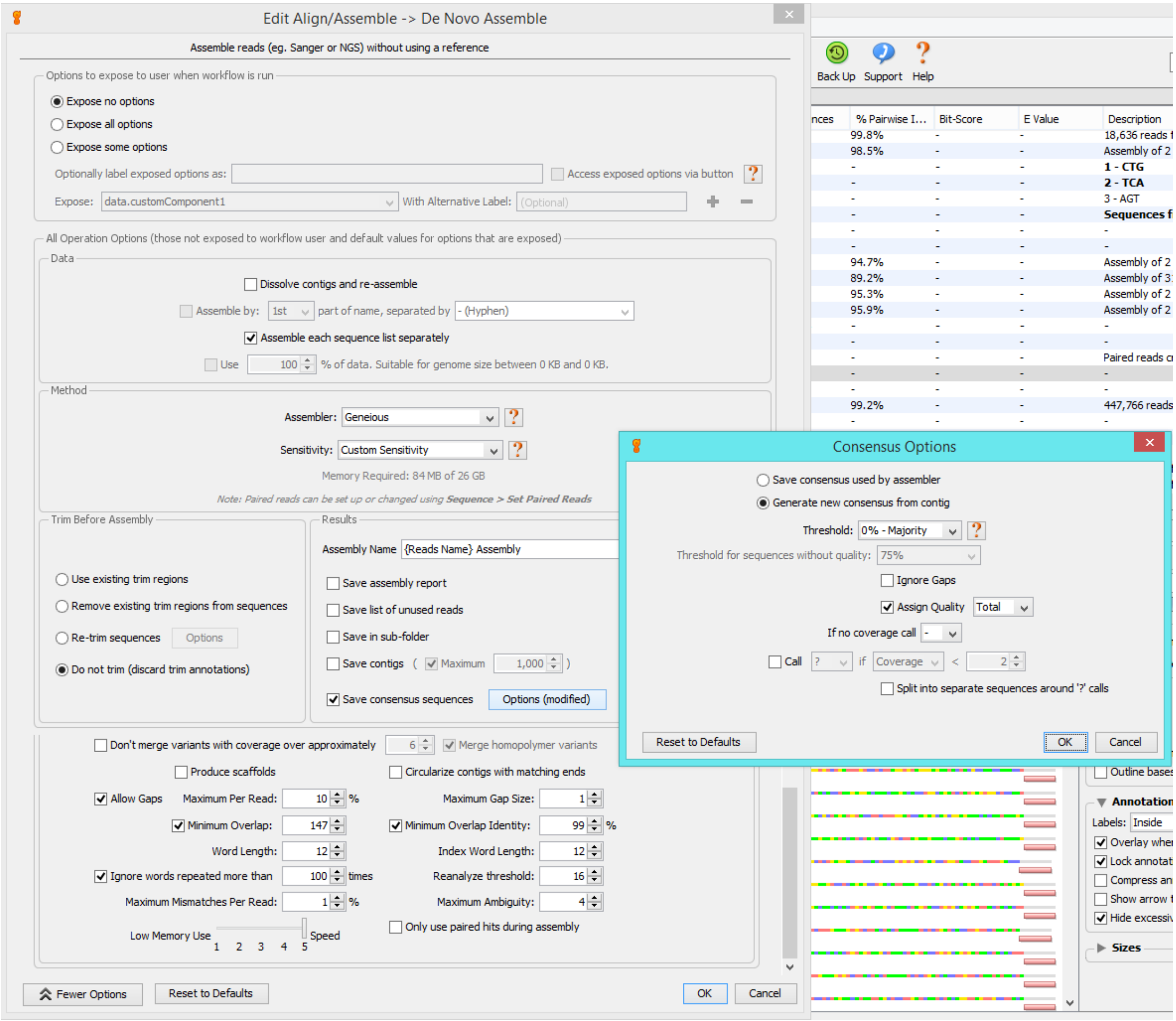

*Word Length and Index Word Length are the parameters that may stronger influence the speed of this operation.

3. Select the two lists of collapsed reads generated by the two previous workflows (i.e. **GenWkf1 + GenWkf2)** and the reference sequence and go to **Workflows > cfDNA GenWkf3**

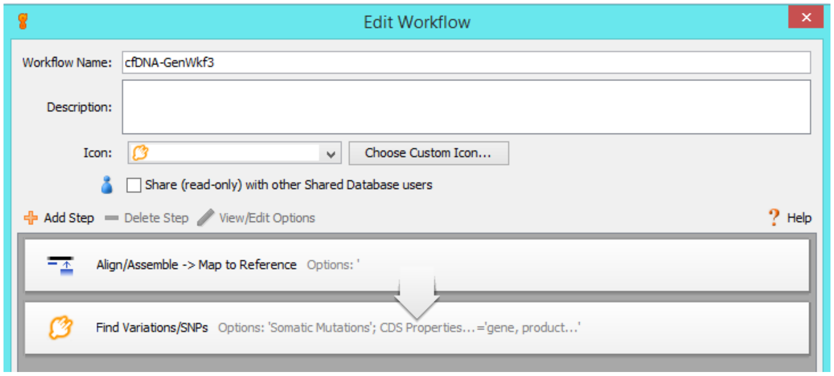

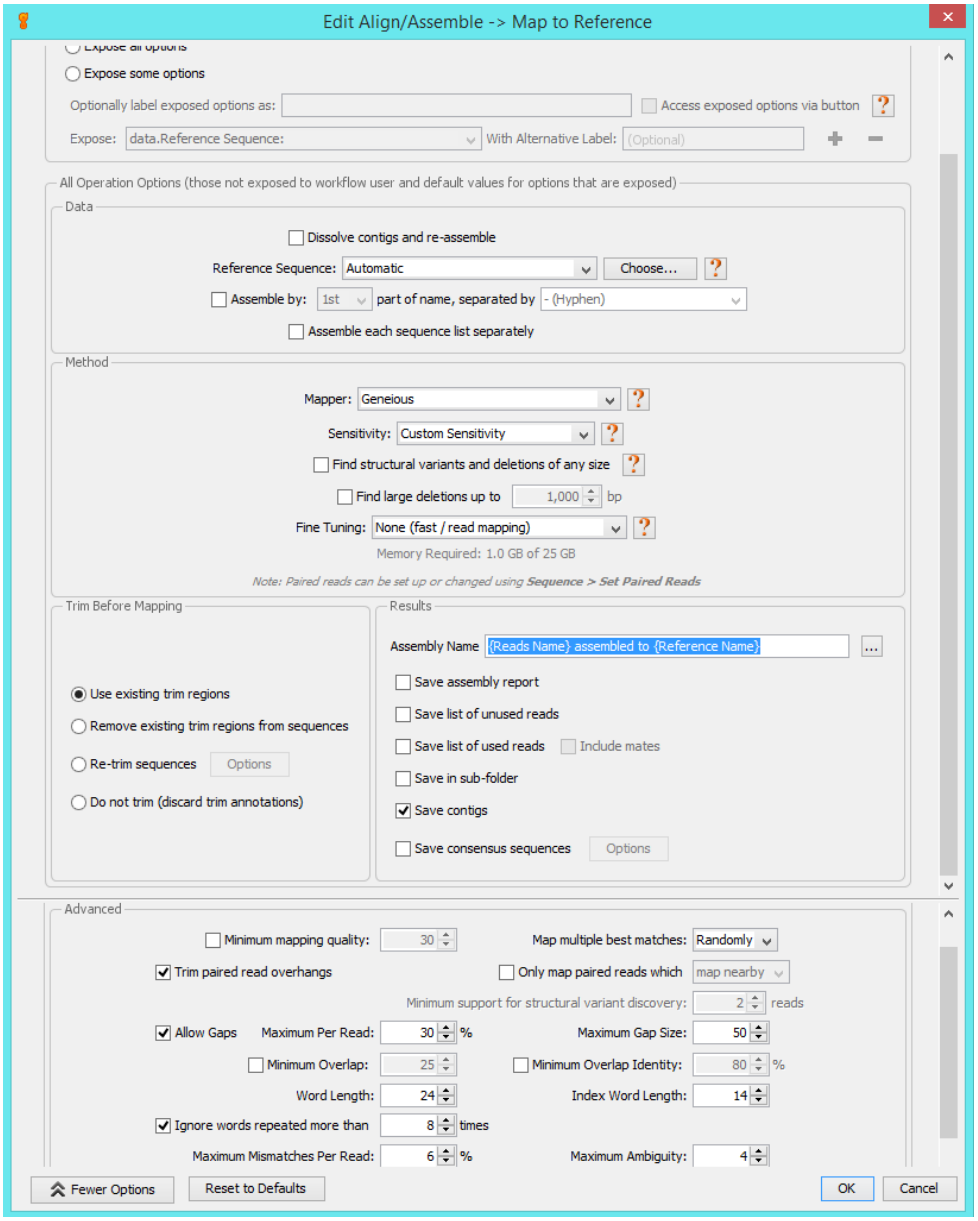

*Customizable options for the *Find Variations/SNPs* step of the workflow

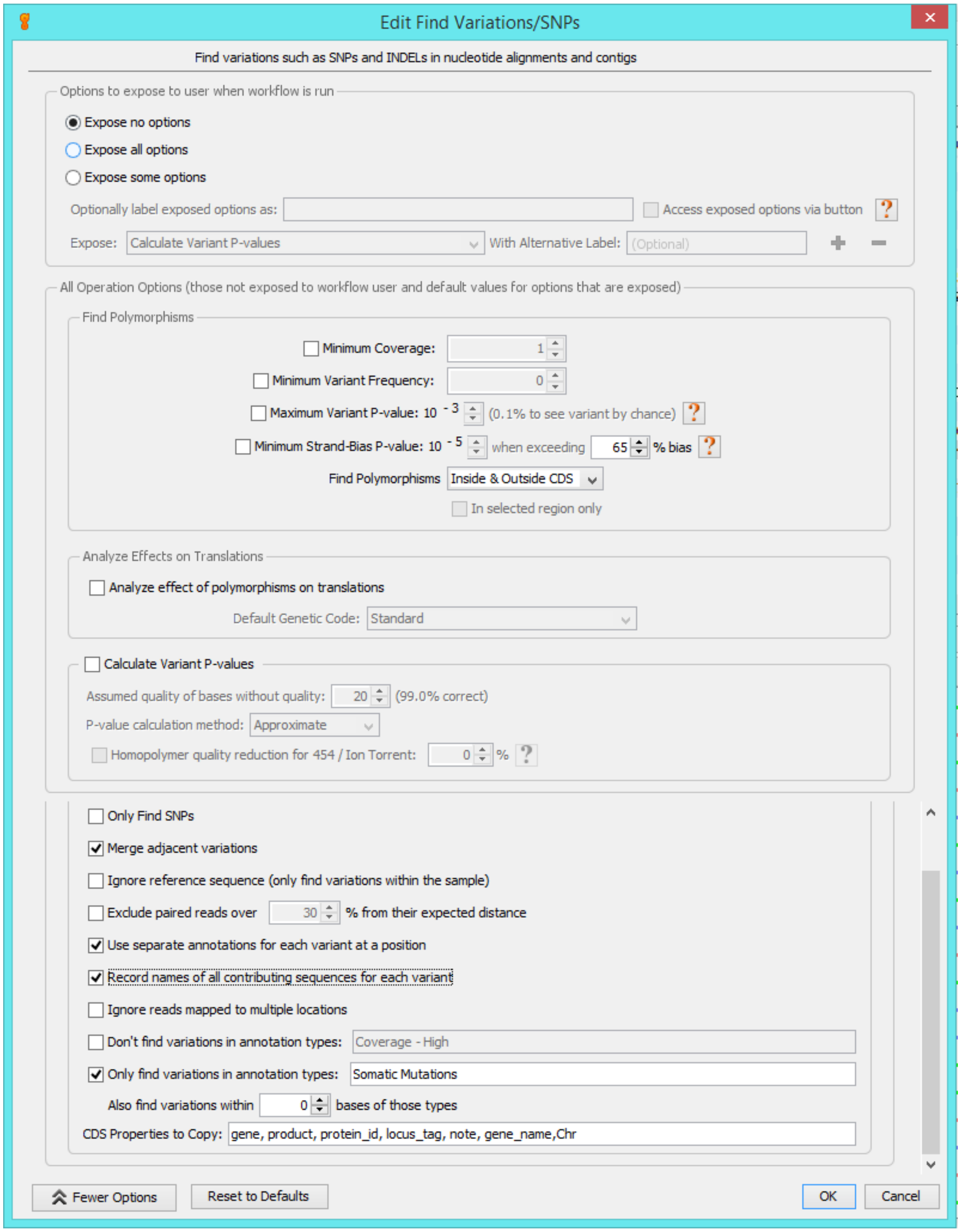

4. Visually inspect for duplex support for ctDNA at a given position. Sort out contigs by the base at a given position to help visualization. Go to the selected base, right-click and select Sort by Bases in column XXX. Be sure to turn off the "Vertically compress contig" option in the advanced menu (right panel).

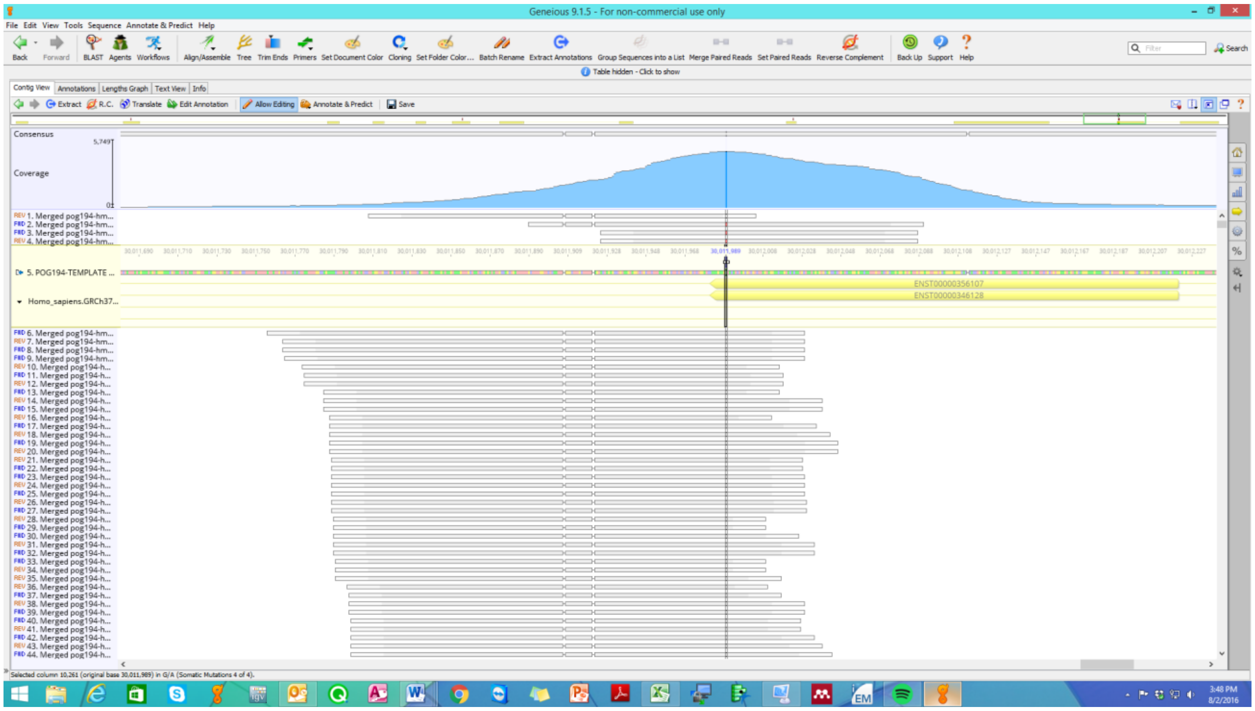

This example shows three ssDNA molecules supporting ctDNA; two of the ssDNA molecules belong to the same duplex as they show different strand-specific tags, map against the reference in different orientations but share the same mapping coordinates and show one single adapter annealing mispairing across the 24-nt semi-degenerate barcodes (see figure below).

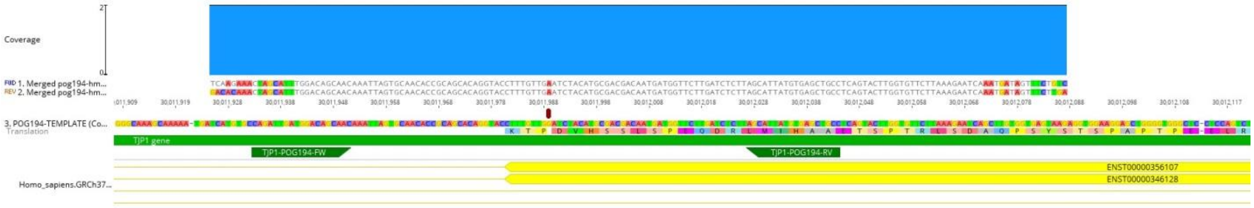

The workflows that analyse sequencing data from enrichment experiments with large gene panels rely on essentially the same algorithms but incorporate a few important modifications. First, there is no minimum number of reads to generate any consensus sequences and non-assembled reads are retained except those filtered out due to low quality. Second, variant calls are not restricted to specific sites and we searched for non-reference alleles across coding regions and splice donor/acceptor sites. The workflows mentioned above can be downloaded

directly from the supplemental material that accompanies this paper. Workflows intended to work on large captured regions have the suffix -lp added to the names of the three main workflows described above.
